# The DEAD-box RNA helicase SHI2 functions in repression of salt-inducible genes and regulation of cold-inducible gene splicing

**DOI:** 10.1093/jxb/erz523

**Published:** 2019-11-20

**Authors:** Bangshing Wang, Haoxi Chai, Yingli Zhong, Yun Shen, Wannian Yang, Junping Chen, Zhanguo Xin, Huazhong Shi

**Affiliations:** 1 Department of Chemistry and Biochemistry, Texas Tech University, Lubbock, TX, USA; 2 School of Life Sciences, Central China Normal University, Wuhan, China; 3 College of Bioscience and Biotechnology, Hunan Agricultural University, Changsha, Hunan, China; 4 Plant Stress and Germplasm Development Unit, USDA-ARS, Lubbock, TX, USA; 5 Lancaster University, UK

**Keywords:** 5' Capping, cold stress, DEAD-box RNA helicase, gene repression, inducible gene, mRNA splicing, polyadenylation, salt stress, SHI2

## Abstract

Gene regulation is central for growth, development, and adaptation to environmental changes in all living organisms. Many genes are induced by environmental cues, and the expression of these inducible genes is often repressed under normal conditions. Here, we show that the *SHINY2* (*SHI2*) gene is important for repressing salt-inducible genes and also plays a role in cold response. The *shi2* mutant displayed hypersensitivity to cold, abscisic acid (ABA), and LiCl. Map-based cloning demonstrates that *SHI2* encodes a DEAD- (Asp-Glu-Ala-Asp) box RNA helicase with similarity to a yeast splicing factor. Transcriptomic analysis of the *shi2* mutant in response to cold revealed that the *shi2* mutation decreased the number of cold-responsive genes and the magnitude of their response, and resulted in the mis-splicing of some cold-responsive genes. Under salt stress, however, the *shi2* mutation increased the number of salt-responsive genes but had a negligible effect on mRNA splicing. Our results suggest that SHI2 is a component in a ready-for-transcription repressor complex important for gene repression under normal conditions, and for gene activation and transcription under stress conditions. In addition, SHI2 also serves as a splicing factor required for proper splicing of cold-responsive genes and affects 5' capping and polyadenylation site selection.

## Teaser

The DEAD-box RNA helix SHI2 represses expression of salt-inducible genes and modulates splicing of cold-responsive genes. SHI2 participates in transcription and post-transcriptional processes in response to stress conditions.

## Introduction

Plants can perceive and respond to stress signals through changes in gene expression patterns. Plant stress responses can be executed at multiple regulatory nodes including transcription, post-transcription, post-translation, and epigenetic modifications (Haak *et al.*, 2017). Many genes involved in stress response and stress adaptation are stress-inducible genes that are commonly repressed under normal conditions but transcriptionally activated by stresses. Previous studies have identified some *cis*-elements and *trans*-acting proteins that are key to the transcriptional activation of stress-inducible genes (Yoshida *et al.*, 2014). However, the molecular mechanism by which these genes are repressed under normal conditions is not well understood.

To better understand the regulation of inducible genes under normal and stress conditions, we developed a screening system and isolated a series of *shiny* (*shi*) mutants with increased expression of the salt-inducible luciferase (LUC) reporter gene under normal and stress conditions (Jiang *et al.*, 2013). Characterization of a few of these mutants has shown that the SHI1–SHI4 complex represses gene transcription possibly by preventing mRNA capping and transition from transcription initiation to elongation (Jiang *et al.*, 2013), while SHI5/HDA6 binds specifically to the promoter regions of some key defense genes, causing histone deacetylation and repression of stress-inducible gene expression (Wang *et al.*, 2017). These studies have suggested that some of the inducible genes are likely to be repressed under non-inducible conditions by a ready-for-transcription repressor complex consisting of general transcription factors, chromatin-modifying enzymes, and protein components for co-transcriptional processes such as 5' capping, mRNA splicing, and polyadenylation.

Post-transcriptional mRNA splicing has been implicated in gene regulation in plant abiotic stress response (Mazzucotelli *et al.*, 2008). However, the involvement of 5' capping and alternative polyadenylation (APA) in plant stress response has rarely been reported. The 5' capping is a process in which a 7-methyguanylate (m^7^G) cap is co-transcriptionally added onto the 5' end of each RNA polymerase II-transcribed mRNA and serves in multiple biological functions: these include its association with the cap-binding complex (CBC) to mediate efficient pre-mRNA splicing, 3' end processing, mRNA exporting, and translation initiation (Flaherty *et al.*, 1997; Daneholt, 2001; Pabis *et al.*, 2013; Ramanathan *et al.*, 2016). APA generates non-canonical mRNA isoforms and affects the fate of transcripts and the functions of the protein. APA plays a role in maintaining the proper growth and development of plants (Simpson *et al.*, 2003; Hunt, 2014) and has been implicated in several human diseases (Shi and Manley, 2015; Chang *et al.*, 2017).

DEAD-box RNA helicase, which derived its name from the conserved motif (Asp-Glu-Ala-Asp, or DEAD), is involved in various molecular processes such as transcription, pre-mRNA splicing, ribosome biogenesis, RNA transport, translation initiation, organelle gene expression, and RNA degradation (Rocak and Linder, 2004; Cordin *et al.*, 2006). In yeast, three DEAD-box RNA helicase proteins SUB2, PRP28, and PRP5 are required for splicing (Staley and Guthrie, 1998). In higher eukaryotes, the RNA helicase P68 is a component of the spliceosome and essential to the constitutive and alternative splicing (Guil *et al.*, 2003), while the RNA helicase P72 is only required for alternative splicing (Hönig *et al.*, 2002). The Arabidopsis genome has a large family of DEAD-box proteins consisting of 58 members (Mingam *et al.*, 2004). Several Arabidopsis DEAD-box RNA helicases have been implicated in plant abiotic and biotic stress responses by affecting specific RNA processing events (Gong *et al.*, 2005; Kant *et al.*, 2007; Kim *et al.*, 2008, Guan *et al.*, 2013). RNA helicase LOS4 regulates mRNA export and plays an essential role in cold stress response in Arabidopsis (Gong *et al.*, 2005), while RCF1, a DEAD-box RNA helicase, is crucial for cold-responsive gene regulation and cold tolerance (Guan *et al.*, 2013). The STRS1 and STRS2 RNA helicases suppress the expression of stress-responsive transcription, and mutations in these two genes rendered the mutant plants more tolerant to salt, heat, and osmotic stresses (Kant *et al.*, 2007). Despite much progress, the mechanisms of DEAD-box RNA helix family proteins in regulating the plant response to various stresses remain largely unknown.

In this study, we identify and characterize the *SHINY2* (*SHI2*) gene encoding a DEAD-box RNA helicase in Arabidopsis. The *shi2* mutant is hypersensitive to ABA, low temperature, and LiCl treatment. Our results indicate that SHI2 is involved in pre-mRNA splicing, 5' capping, and 3' processing, and plays an important role in the repression of salt-inducible genes and modulation of cold-responsive gene splicing.

## Materials and methods

### Plant materials and growth conditions

The Arabidopsis (*Arabidopsis thaliana*) accessions Col-0 and Landsberg *erecta* (Ler) were used in this study. The transgenic line with the expression of the firefly LUC reporter gene driven by the promoter of a salt-inducible gene *SOT12* (At2G03760) in the Col-0 background (SOT12:LUC) was used as the wild type. The *shi2* mutant characterized in this study contains a single copy of the SOT12:LUC transgene in the Col-0 background and is one of the *shiny* mutants, *shiny2*, isolated as previously described (Jiang *et al.*, 2013). The T-DNA insertion mutant line with a T-DNA insertion in the At1G20920 gene (SALK_146567) was obtained from the Arabidopsis Biological Resource Center (ABRC). The homozygous mutant of the T-DNA line was identified by PCR-based genotyping and used for genetic complementation.

For all plate-based assays/experiments, the Arabidopsis seeds were surface-sterilized, planted on the agar plates, and then incubated at 4 °C for 2 d before being transferred to a growth chamber set to normal growth conditions of 22 °C and a 16 h/8 h light to dark cycle. For the cotyledon greening assay, ~50 seeds were sown on half-strength Murashige and Skoog (1/2 MS) medium with 0.6% agar supplemented with different concentrations of ABA. A green seedling was defined as having two obviously green cotyledons. For the primary root growth assay, Arabidopsis seeds were planted on 1/2 MS medium with 1.2% agar and allowed to grow in a vertical position for ~5 d. The seedlings were then gently transferred onto plates containing the medium supplemented with different concentrations of chemicals and allowed to grow vertically for another 10 d.

### Map-based cloning and complementation

The *shi2* mutant was crossed with the Ler ecotype, and the resulting F_1_ was self-pollinated to generate F_2_ progeny. The 5-day-old plate-grown F_2_ seedlings were evaluated for their luminescence levels, and individuals with higher luminescence were selected and used for map-based cloning of the *SHI2* gene. For the molecular complementation assay, the ORF of the *SHI2* (At1G20920) sequence was first cloned into the pENTR1A gateway entry vector, followed by recombining the ORF sequence into the destination vector pEarleyGate100 using the Gateway LR Clonase II Enzyme Mix (Invitrogen, Carlsbad, CA, USA). The resulting construct was introduced into *Agrobacterium tumefaciens* GV3101 and then transferred to the *shi2* mutant using the *Agrobacterium*-mediated floral dip transformation method (Clough and Bent, 1998). Primers used for cloning are listed in Supplementary Table S1 at *JXB* online. For genetic complementation, the T-DNA mutant allele was crossed with *shi2*, and the F_1_ seedlings were subjected to LUC imaging.

### Subcellular localization and promoter–GUS analysis

To determine the subcellular localization of SHI2, the *SHI2* gene in the pENTR1A:SHI2 entry vector was recombined into pMDC43 to generate an SHI2–green fluorescent protein (GFP) fusion construct using the Gateway LR Clonase II Enzyme Mix (Invitrogen). The construct was transformed into Arabidopsis Col-0 wild type by the floral dip method (Clough and Bent, 1998). The GFP signals in the roots of transgenic plants were examined by using the Olympus IX81 inverted laser scanning confocal microscope system at an excitation of 488 nm and emission of 525 nm.

For promoter–β-glucuronidase (GUS) analysis, a 3527 bp promoter fragment of *SHI2* upstream of the translation initiation codon ATG was cloned into pCAMBIA1381Z. The construct was then introduced into Arabidopsis Col-0 by using the floral dip method, and homozygous transgenic T_2_ plants were identified, stained with X-Gluc staining buffer (10 mM Tris pH 7.0, 10 mM EDTA, 0.1% Triton X-100. and 2 mM 5-bromo-4-chloro-3-indolyl-β-d-glucuronic acid) for 12–24 h at 37 °C, and examined for the expression of the *SHI2* gene in different tissue types of Arabidopsis plants.

### Detection of stress-inducible gene expression and intron retention by RT–qPCR

Seven-day-old seedlings of the wild type and *shi2* mutant grown in 1/2 MS agar (0.6%) medium under normal conditions (control) were used for cold, ABA, and NaCl treatments. Control and treated seedlings were collected for RNA extraction using the Plant RNA Purification Reagent (Invitrogen). Reverse transcription (RT) for cDNA synthesis was performed by using the TransScript One-Step gDNA Removal and cDNA Synthesis SuperMix kit (TransGen Biotech, Beijing, China). Resulting cDNAs were used as templates for quantitative PCR (qPCR) with Sybr Green qPCR SuperMix (TransGen Biotech, Beijing, China). The qPCR was performed with a CFX96 real-time PCR detection system (Bio-Rad, Hercules, CA, USA). ACT7 was used as an internal control. All the reactions were performed in triplicate. The primers used are listed in Supplementary Table S2.

For the intron retention assay, 7-day-old seedlings of the wild type and *shi2* mutant were transferred to 4 °C for cold treatment of 0, 3, 6, and 12 h. The whole seedlings were then harvested for RNA extraction. To determine the intron retention in RNA samples, primers (Supplementary Table S2) were designed to amplify the partial sequence of the first intron and the adjacent exon of the target genes by using RT–qPCR as described above. The transcript levels were further normalized against the 0 h control of wild-type samples.

### Transcription activation assay

Different portions of the *SHI2* cDNA sequence were amplified by PCR. The sequences were cloned into the pENTR1A vector and then recombined into the destination vector pGBKT7. The constructs were then transformed into yeast strain Y190. Yeast cells grown on a synthetic medium without tryptophan were subjected to β-galactosidase assay for the self-activation test.

The reporter–effector system used in this study was described previously (Ulmasov *et al*., 1995, 1997; Tiwari *et al*., 2001, 2003; Chinnusamy *et al.*, 2003). The coding sequence for the SHI2c protein from amino acids 875 to 1167 was cloned into the reporter vector. Then both the reporter vector and effector vector (35S-GAL4 BD) were co-transformed into Arabidopsis protoplasts following the method described by Yoo *et al.* (2007). ARF5M (Auxin Response Factor 5 Middle region) (Tiwari *et al.*, 2003) was used as a positive control for transcriptional activation analysis.

### Detection of mRNA capping

5' RACE was used to analyze the capping ratio of the LUC mRNAs according to the method established by Jiang *et al.* (2013). Briefly, 2 μg of total RNA was reverse transcribed into cDNA. The cDNA was added with a poly(A) tail at its 5' end using terminal transferase (New England Biolabs) which served as a template for a series of nested PCRs using the adaptor primer and gene-specific primers. The PCR products were then cloned into the pGEM-T Easy vector (Promega, Madison, WI, USA), and 20–40 positive clones were isolated and sequenced. The additional G at the very end of the 5' terminus of each cDNA was counted as a capped mRNA.

The Arabidopsis eIF4E–glutathione *S*-transferase (GST) recombinant protein was used to separate capped and uncapped mRNA from total RNA. Recombinant protein was expressed in *Escherichia coli* and purified. Then 20 µg of total RNA was incubated with the purified eIF4E–GST protein, and capped and uncapped mRNAs were separated as described by Jiang *et al.* (2013). Both capped and uncapped mRNAs were analyzed by using RT–qPCR. The primers used in this study are listed in Supplementary Table S3.

### Determination of polyadenylation sites

The polyadenylation sites of the mRNAs were determined by using 3' RACE as described by Jiang *et al.* (2013). The cDNA was reverse transcribed from total RNA using avian myeloblastosis virus (AMV) reverse transcriptase (Promega), and oligo(dT)_17_ primer. The cDNA was then utilized as the template for nested PCR by using the adaptor primer and gene-specific primer. PCR products were cloned into pGEM-T Easy vector. At least 20 independent clones were sequenced, and the polyadenylation sites were scored according to the sites linking with the poly(A). The primers used are listed in Supplementary Table S3.

### RNA-seq analysis

RNA sequencing (RNA-seq) and RT–qPCR were conducted according to Shen *et al.* (2016). Briefly, 10-day-old wild-type and *shi2* seedlings grown under normal conditions were randomly assigned into three groups: (i) control; (ii) cold treated at 4 °C for 12 h; and (iii) salt stressed in 100 mM NaCl for 6 h. Samples were harvested at the end of each treatment and total RNA was isolated using the RiboPure™ RNA Purification Kit (Invitrogen) according to the manufacturer’s protocol. Libraries were constructed, quantified, and quality checked with an Agilent 2100 Bioanaylzer, and then sequenced using an Illumina HiSeq 2500 System platform. Clean sequencing reads were mapped to the Arabidopsis genome (TAIR10 release) using the TopHat program (version 2.0.8) (Trapnell *et al.*, 2009). Differentially expressed genes (DEGs) were identified by edgeR (version 3.4.2) (Robinson *et al.*, 2010) with cut-off as fold change >2 and false discovery rate (FDR) <0.05.

The differential exon expression was analyzed using DEXseq (Anders *et al.*, 2012). This analysis calculated the number of reads for each exon in each sample. Based on these data, the expression level of a certain exon (X) from a certain gene (Y) was calculated by comparing the number of exon X containing Y transcripts with the total number of all Y transcripts. The exons differentially expressed under different conditions, and the genes containing the differentially expressed exon were then identified by comparing the exon expression level data of different samples.

For validation of the RNA-seq results by RT–qPCR, total RNA was extracted from different samples using the Plant RNA Purification Reagent (Invitrogen). A 2 µg aliquot of total RNA was reverse transcribed with random primers using AMV reverse transcriptase (Promega). The resulting cDNAs were used as templates for real-time PCR with LightCycler^®^ 480 master mixes (Roche, Penzberg, Germany). *Actin2* was used as an internal control. The primers used are listed in Supplementary Table S4. Three biological replicates were used, and the data are shown as the average ±SD of three biological replicates.

## Results

### Phenotyping of the *shi2* mutant

**Fig. 1.**
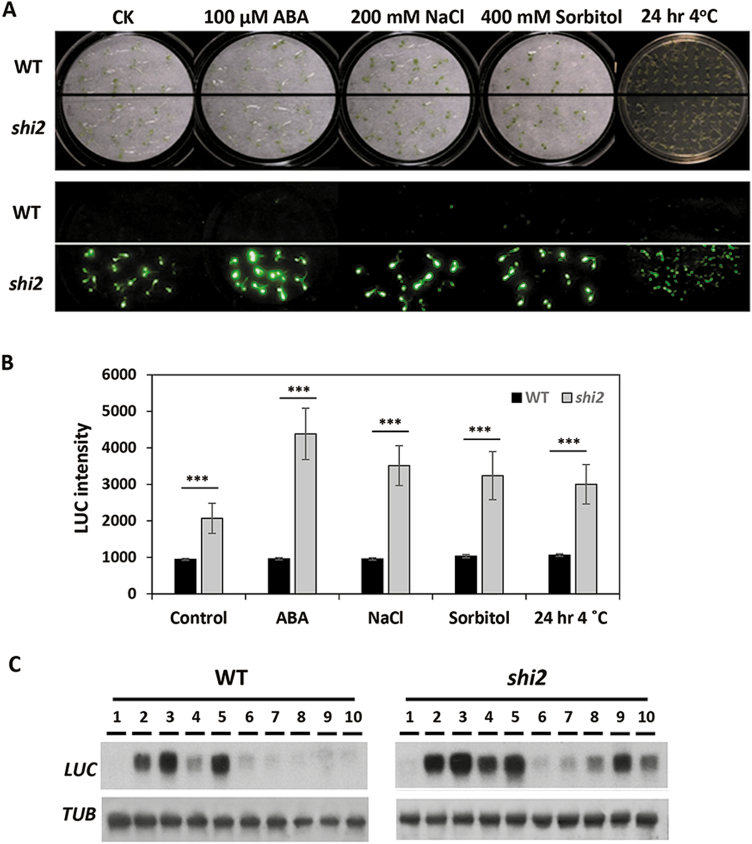
The *shi2* mutant shows higher expression of the *LUC* gene. (A) Bioluminescence images of the wild type and *shi2* mutant under different stress conditions. Seven-day-old seedlings grown on 1/2 MS medium were subjected to different stress conditions: 100 µM ABA for 3 h, 200 mM NaCl for 5 h, 400 mM sorbitol for 5 h, and 4 °C cold for 24 h. The upper panel shows bright field images and the lower panel shows bioluminescence images. (B) Quantitative analysis of bioluminescence intensities in wild-type and *shi2* seedlings with or without stress treatments. Asterisks indicate a statistically significant difference (*n*=15, ***P<0.001 by Student’s *t*-test). (C) Northern blot showing the transcript levels of *LUC* genes in wild-type and *shi2* seedlings in response to different stress conditions: 1, control; 2, 4 °C for 6 h; 3, 4 °C for 12 h; 4, 100 μM ABA for 30 min; 5, 100 μM ABA for 3 h; 6, 100 mM NaCl for 6 h; 7, 100 mM NaCl for 12 h; 8, 200 mM NaCl for 6 h; 9, 200 mM NaCl for 12 h; 10, 400 mM sorbitol for 5 h. (This figure is available in color at *JXB* online.)

The *shi2* mutant displayed no morphological or developmental defects under normal conditions but showed enhanced expression of the LUC reporter gene under both normal and stress conditions (Fig. 1). Figure 1A shows the LUC imaging of the wild-type and *shi2* plants under ABA, NaCl, sorbitol, and cold treatments. Quantification of the LUC activities showed higher LUC expression in the *shi2* mutant than in the wild type under normal and stress conditions (Fig. 1B). Northern blot analysis confirmed that the expression of the *LUC* gene was indeed induced to higher levels in *shi2* than in the wild type after stress treatments (Fig. 1C).

Genetic analysis showed that all F_1_ seedlings had LUC activity similar to that of the wild type, and the F_2_ population showed a 3:1 segregation ratio of the wild type to the *shi2* mutant (220:68; χ ^2^=0.30) for bioluminescence phenotype. This result indicated that *shi2* is a recessive mutation at a single nuclear locus.

**Fig. 2.**
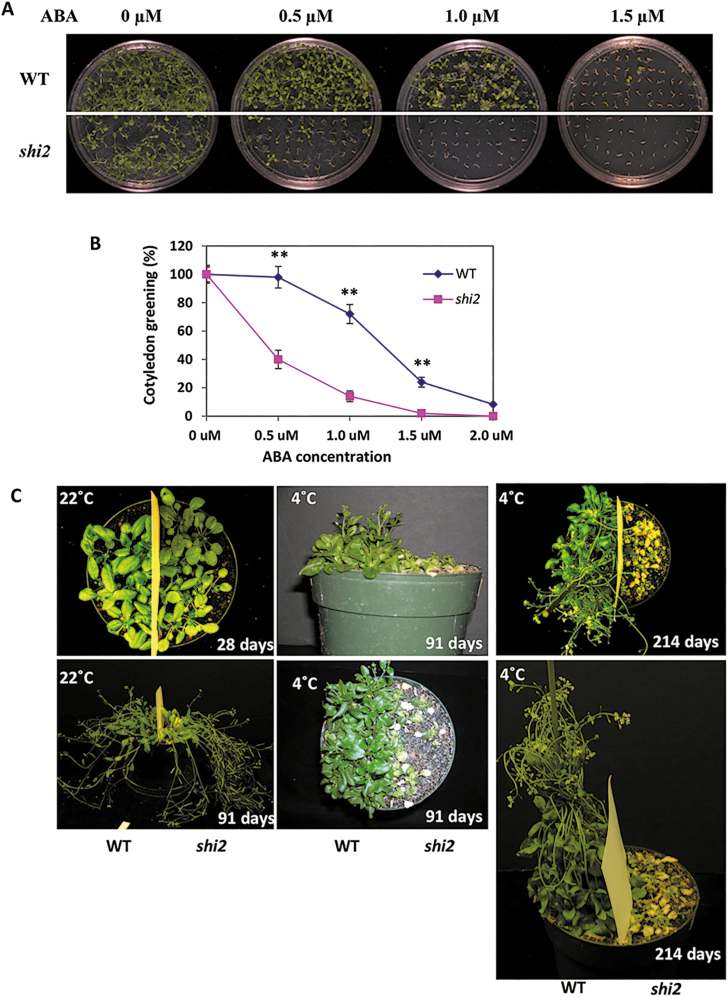
*shi2* is hypersensitive to ABA and shows a growth defect under cold conditions. (A) Images showing seed germination of the wild type and *shi2* mutant in 1/2 MS medium containing different concentrations of ABA. (B) Quantitative measurement of cotyledon greening at 12 d after seed germination. Asterisks indicate a statistically significant difference; the same experiment was repeated three times (*n*=3, **P<0.01 by Student’s *t*-test). (C) Images showing increased growth inhibition of the *shi2* mutants under cold stress conditions. Seven-day-old seedlings grown in 1/2 MS agar medium were transferred to soil and grown for an additional 10–14 d under normal growth conditions. These plants were then placed in a 4 °C cold room for 91 d and 214 d with continuous light. (This figure is available in color at *JXB* online.)

In addition to the bioluminescence phenotype (Fig. 1A), the s*hi2* mutant was more sensitive to ABA than the wild type during seed germination (Fig. 2A) and showed significantly more reduction in cotyledon greening (~40%) than the wild type (~98%) at 0.5 µM ABA (Fig. 2B). Furthermore, the *shi2* mutant displayed severe growth inhibition under low-temperature stress (Fig. 2C). In contrast to the hypersensitive response of *shi2* to cold stress, no differences were observed between *shi2* and wild-type root growth under different NaCl and mannitol treatment conditions (Supplementary Fig. S1).

### *SHI2* encodes a DEAD-box RNA helicase

**Fig. 3.**
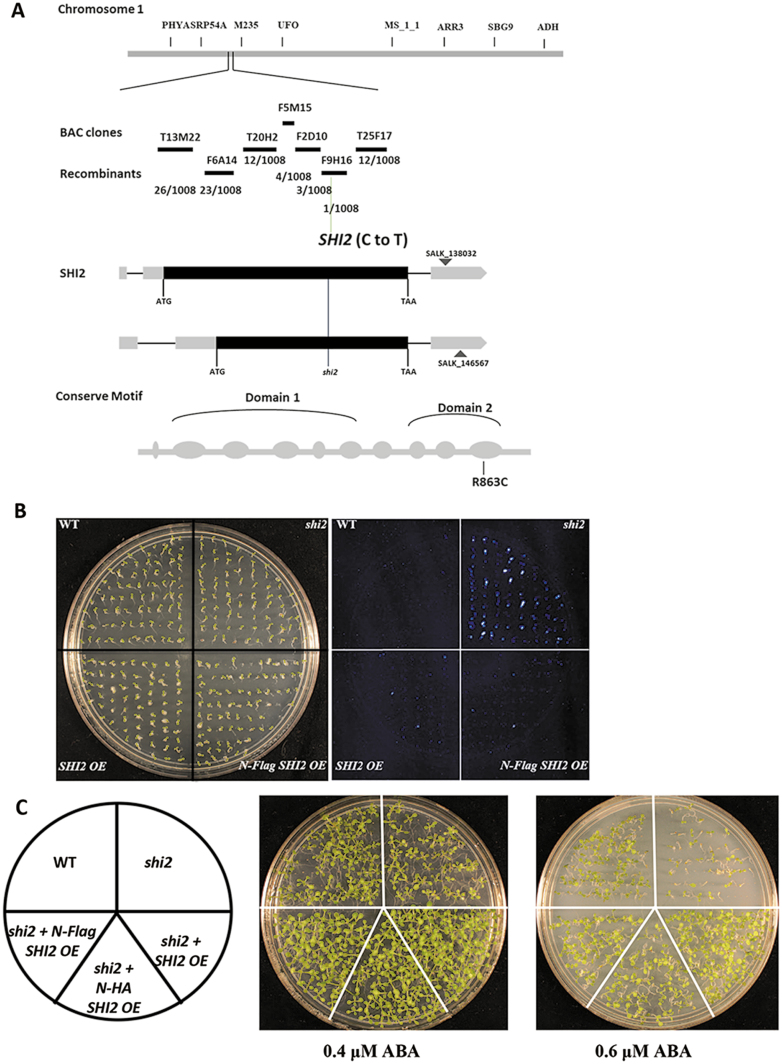
Map-based cloning and complementation of the *shi2* mutant. (A) Map-based cloning of the *SHI2* locus. A change of C to T at nucleotide position 2587 and its resulting amino acid substitution in the *shi2* mutant are shown. (B) Molecular complementation of the *shi2* mutant by the wild-type *SHI2* gene. A bright field image of seedlings grown on a 1/2 MS plate is shown in the left panel and the corresponding bioluminescence image is shown in the right panel. (C) Complementation of the *shi2* mutant by overexpression of SHI2. Seeds were plated for germination assay with 0.4 µM and 0.6 µM ABA, and photographs were taken after 14 d. (This figure is available in color at *JXB* online.)

The *shi2* mutation was first mapped to an 80 kb region on chromosome 1 between the markers F2D10 and F9H16. Detailed sequencing analysis identified a single nucleotide substitution of C to T in the gene At1G20920 (Fig. 3A). To validate that At1G20920 was indeed the *SHI2* gene, we selected a T-DNA line (SALK_146567) with an insertion at the 3'-untranslated region (UTR) of the At1G20920 gene and made a complementing cross between *shi2* and the T-DNA line. The resulting F_1_ progeny showed the same enhanced LUC imaging phenotype as that of *shi2* (Fig. 3A; Supplementary Fig. S2), indicating that the T-DNA line is allelic to the *shi2* mutant. Furthermore, molecular complementation showed that the *shi2* mutant expressing the wild-type *SHI2* gene exhibited phenotypes comparable with those of the wild type, including the LUC imaging phenotype (Fig. 3B), cotyledon greening in the presence of ABA (Fig. 3C), and sensitivity to LiCl (Supplementary Fig. S3). These results indicate that the At1G20920 is the *SHI2* gene, and the C to T substitution in *SHI2* is the causal mutation for the *shi2* mutant phenotypes.

*SHI2* was annotated to be an intronless gene, encoding a DEAD-box RNA helicase (Fig. 3A). The C to T change in At1G20920 resulted in the substitution of a positively charged polar arginine by a non-charged polar cysteine at position 863 in the last conserved motif HRxGRxGR in the SHI2 protein (Fig. 3A: Supplementary Fig. S4A).

### The *shi2* mutation affects the expression of stress-inducible genes

**Fig. 4.**
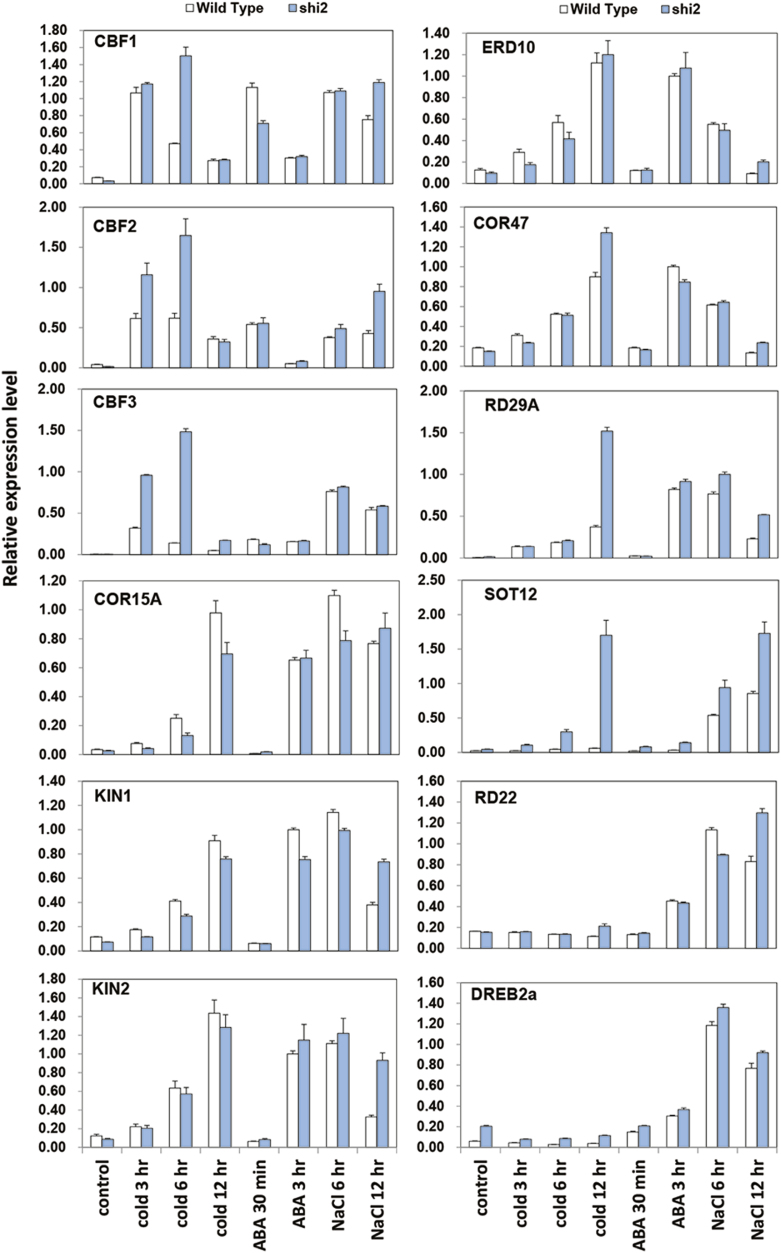
Expression of stress-inducible genes under different stress conditions in the *shi2* mutant and the wild type. Seven-day-old seedlings cultured under normal conditions were treated without (Control) or with the following treatments: 4 °C cold for 3, 6, and 12 h, 100 µM ABA for 30 min and 3 h, or 200 mM NaCl for 6 h and 12 h. The transcript levels were determined by RT–qPCR. Data are means ±SD (*n*=3).

We determined the transcript levels of some well-known stress-inducible genes in the *shi2* mutant and wild type (Fig. 4). The cold-induced expression levels of all three *CBF* genes were markedly higher in *shi2* than in the wild type after a 6 h cold treatment, and *CBF2* and *CBF3* genes, but not the *CBF1* gene, were also induced to higher levels in *shi2* than in the wild type after a 3 h cold treatment. Moreover, the expression of the *CBF* genes under cold was maintained at high levels for longer in *shi2* than in the wild type. However, expression of several other cold-inducible genes, including *Cor15A*, *Cor47*, *KIN1*, *KIN2*, and *ERD10*, did not differ between *shi2* and the wild type. Interestingly, after a 12 h treatment, *RD29A* and *SOT12*, whose expression was not induced by cold in the wild type, were highly induced in *shi2*. Under salt stress, the salt-inducible expression of the *SOT12* gene was clearly higher in *shi2* than in the wild type, as expected, and higher induction of *KIN1*, *KIN2*, *RD29A*, and *RD22* in *shi2* was also observed after a 12 h salt treatment. The expression levels of all the genes shown in Fig. 4 were comparable between *shi2* and the wild type in response to ABA treatment. Together, these results indicate that SHI2 regulates cold and salt stress-responsive genes in Arabidopsis.

### SHI2 is required for pre-mRNA splicing

**Fig. 5.**
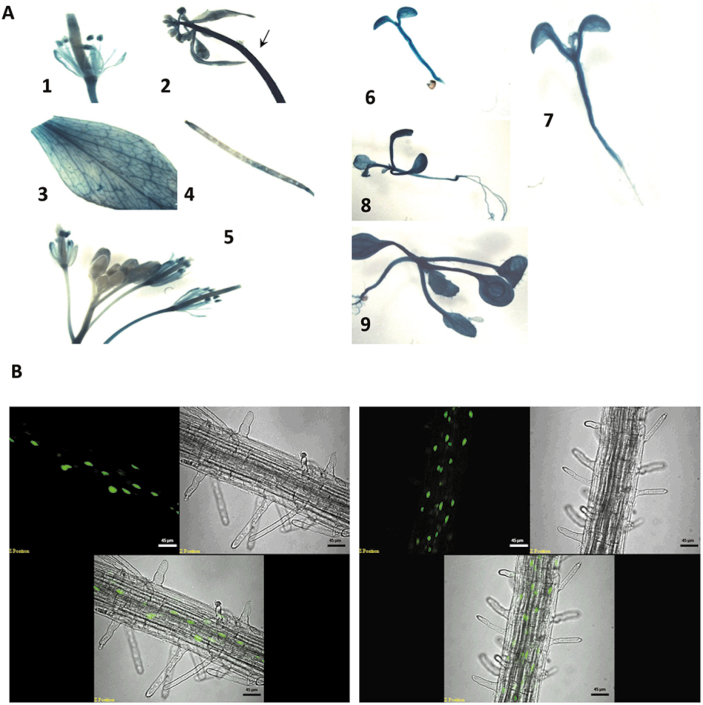
Gene expression and subcellular localization of SHI2. (A) GUS staining showing the expression of the *SHI2* promoter–GUS at different developmental stages and in different organs. 1, Flower; 2, hypocotyl; 3, leaf; 4, silique; 5, meristem/flower; 6, 3-day-old seedling; 7, 7-day-old seedling; 8, 10-day-old seedling; 9, 14-day-old seedling. (B) Confocal images showing the subcellular localization of the SHI2–GFP fusion protein. Scale bars=45 μm. (This figure is available in color at *JXB* online.)

**Fig. 6.**
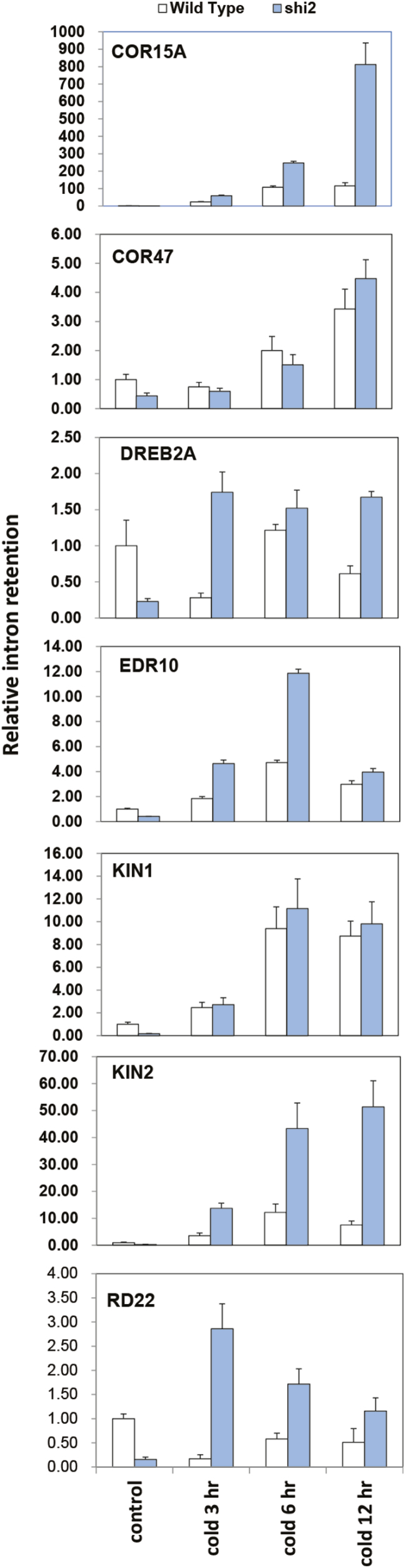
Detection of intron retention in the *shi2* mutant and wild type. Seven-day-old seedlings grown under normal conditions were treated without (Control) or with 4 °C cold for 3, 6, or 12 h. The transcript levels were determined by RT–qPCR using the primers from the first intron and the adjacent exon of the genes. Data are means ±SD (*n*=3). (This figure is available in color at *JXB* online.)

The expression of the *SHI2* gene in plants was analyzed through promoter–GUS analysis, and the results showed a ubiquitous expression of *SHI2* in all tissues tested (Fig. 5A). SHI2–GFP fusion analysis revealed that SHI2 is localized in the nucleus (Fig. 5B). Protein sequence analysis revealed that Arabidopsis SHI2 shares 41% similarity with the yeast *Saccharomyces* pre-RNA processing 5 (PRP5). Both SHI2 and PRP5 are highly conserved at the RNA helicase signature motifs (Supplementary Fig. S4A). The yeast *PRP5* is a well-studied gene involved in spliceosome assembly and is required for RNA splicing in yeast cells (Dalbadie-McFarland and Abelson, 1990). SHI2 may function in Arabidopsis similarly to PRP5 in yeast. Northern blot analysis showed that the cold-inducible gene *COR15A* had an additional band of a larger size of mRNA transcript after cold treatment (Supplementary Fig. S5A), indicating possible mis-splicing of pre-mRNA in the *shi2* mutant. Subsequently, we detected intron retention in *COR15A* and *COR6.6/KIN2* transcripts and found that the *shi2* mutant accumulated substantially more intron-retaining mRNAs than the wild type under cold stress conditions (Supplementary Fig. S5B, C). Intron retention was further analyzed by RT–qPCR, and the results showed that, in addition to *COR15A* and *KIN2*, *EDR10* and *RD22* genes also accumulated more intron-containing mRNA in *shi2* than in the wild type after cold stress treatment (Fig. 6). These results indicate that SHI2 is required for proper mRNA splicing of some of the cold-responsive genes.

**Fig. 7.**
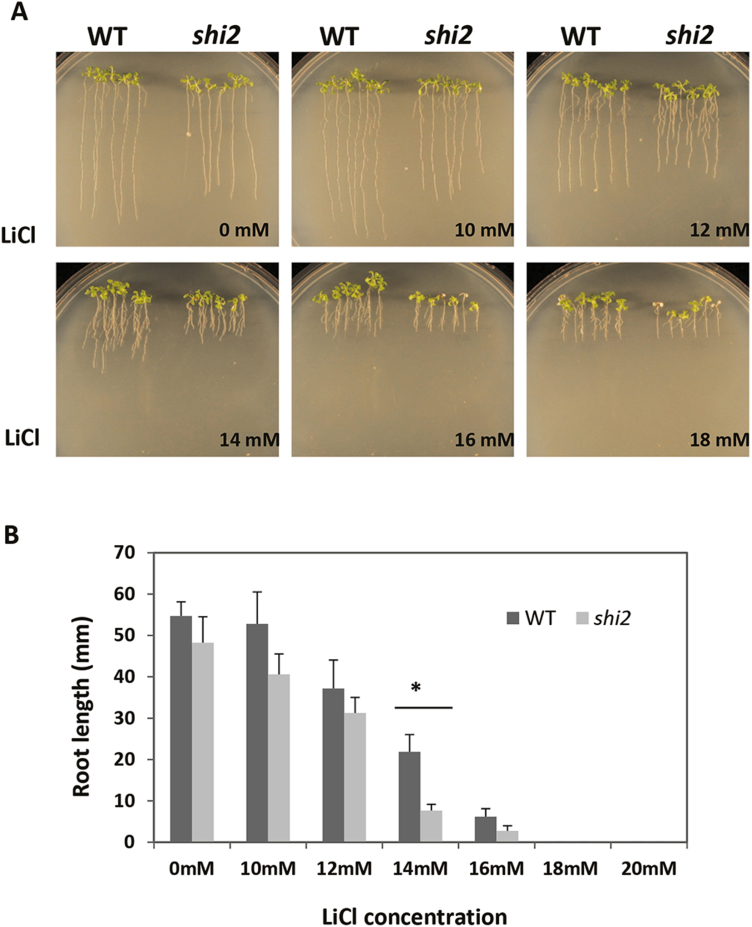
The *shi2* mutant is more sensitive to LiCl than the wild type. (A) Root growth of the *shi2* mutant and wild type in the presence of different concentrations of LiCl. (B) Quantitative measurement of primary root growth. Primary root length was measured on the 10th day after transfer to media containing different concentrations of LiCl. Data are means ±SD (*n*=15). Asterisks indicate a statistically significant difference (*P<0.05 by Student’s *t*-test). (This figure is available in color at *JXB* online.)

The *shi2* mutant also displayed hypersensitivity to LiCl compared with the wild type (Fig. 7A). LiCl inhibits RNA processing enzymes, and yeast mutants defective in RNA splicing show hypersensitivity to LiCl (Dichtl *et al.*, 1997). Primary root growth of the *shi2* mutant was inhibited more by 14 mM LiCl than the wild type (Fig. 7B). Hence, our findings here suggest that SHI2 may be required for pre-mRNA splicing of some cold-inducible genes, possibly as an essential component of a splicing complex.

### The C-terminal region of SHI2 can act as a transcription activator

**Fig. 8.**
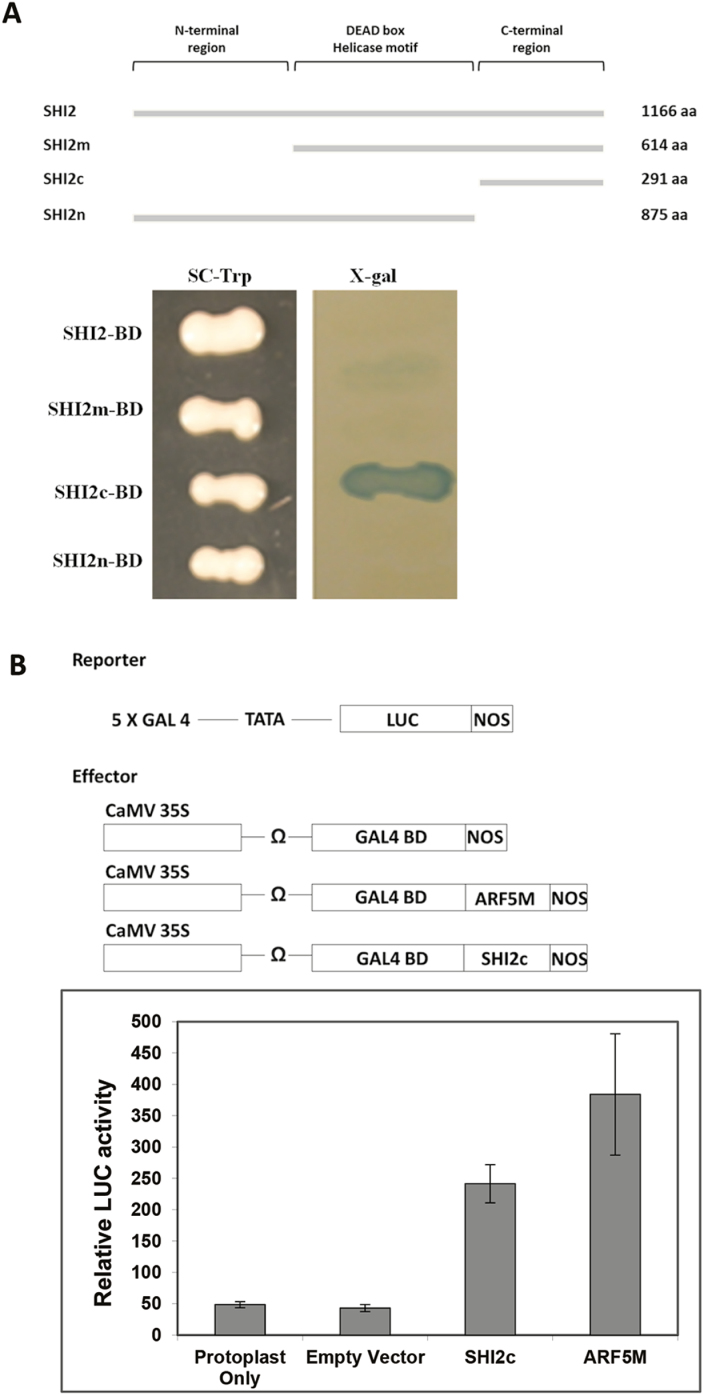
Transcriptional activation by the C-terminus of SHI2. (A) Activation of the reporter gene *β*-*Gal* by SHI2c in yeast. Different forms of SHI2 were fused with the DNA-binding domain (BD) in the bait construct, and self-activation assay was used to determine the transcription activation activity of SHI2, SHI2m, SHI2c, and SHI2n. (B) SHI2c shows much higher LUC activity than the empty vector control in Arabidopsis protoplasts. ARF5M (Auxin Response Factor 5 Middle region) was used as a positive control. Data are means ±SD (*n*=3). (This figure is available in color at *JXB* online.)

To test whether SHI2 transcriptionally regulates gene expression, we fused different regions of SHI2 with a DNA-binding domain in the yeast bait construct. Interestingly, the C-terminal region of SHI2 (SHI2c) activated the reporter gene (*β-Gal*) expression in yeast cells but the other three regions did not (Fig. 8A). This result suggests that SHI2 can be associated with the transcriptional machinery through its C-terminus to modulate gene transcription in yeast. To further confirm the function of SHI2 in gene transcriptional regulation in plants, we performed transcription activation assays in plant cells as described previously (Ulmasov *et al*., 1995, 1997; Tiwari *et al*., 2001, 2003; Chinnusamy *et al.*, 2003). The protoplasts with the SHI2c fusion protein displayed significantly higher LUC activity than those with the empty vectors (Fig. 8B). As a positive control, ARF5M, which was defined as an activation domain (Tiwari *et al.*, 2003), also showed transcriptional activation activity in Arabidopsis. These results further support that SHI2 could function as a transcriptional regulator in plant cells.

### SHI2 is involved in mRNA capping and polyadenylation site selection

**Fig. 9.**
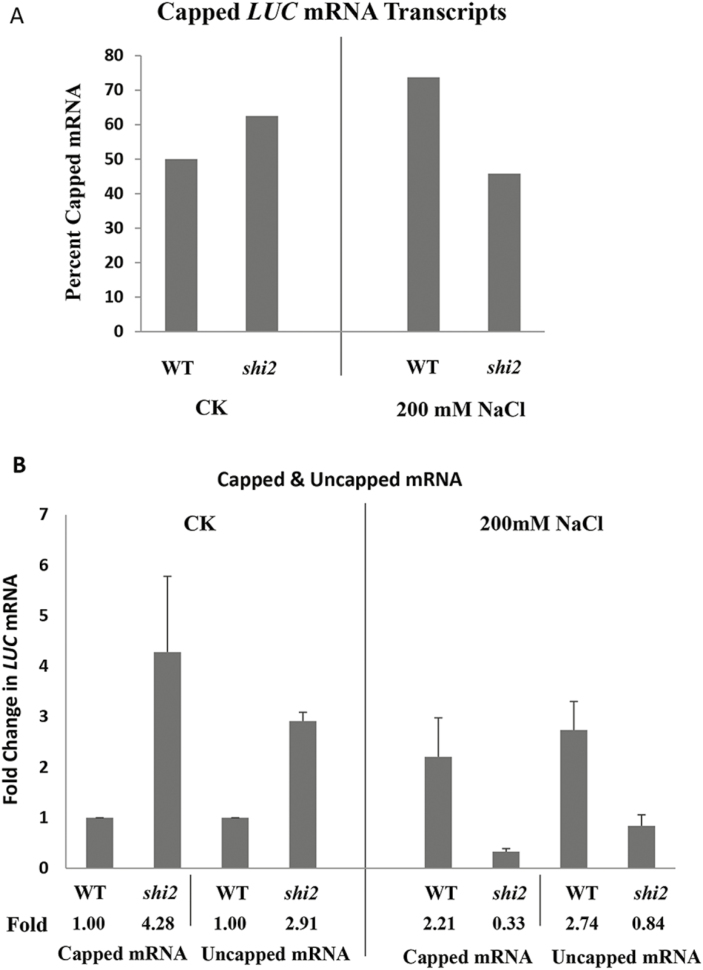
The *shi2* mutation alters the capping pattern of the *LUC* mRNA. (A) Quantification of 5' capping of *LUC* mRNA using 5' RACE. The 5' cap was determined by the presence of a G at the very 5' end of the cDNA, which is not a genomic nucleotide in the *LUC* gene at this position. (B) Detection of 5' capping of *LUC* mRNA using the cap-binding protein eIF4E. The levels of capped and uncapped mRNAs were determined by RT–qPCR. Data are means ±SD (*n*=3). The salt treatment was 200 mM NaCl for 5 h.

To test if SHI2 functions similarly to SHI1 and SHI4 (Jiang *et al.*, 2013) in mRNA 5' capping and polyadenylation site selection, we quantified the capped and uncapped *LUC* mRNA transcripts in the wild type and *shi2* mutant under both normal and stressed conditions. The results showed that among the total *LUC* mRNA, the wild type had ~52% capped *LUC* mRNA transcripts while *shi2* had ~62% under normal growth conditions (Fig. 9A). Under salt stress (200 mM NaCl for 5 h), capped *LUC* mRNA transcripts in the wild type increased to 74% while they were significantly decreased to 46% in the *shi2* mutant.

The 5' capping was further analyzed by using the Arabidopsis cap-binding protein eIF4E, as described in Jiang *et al.* (2013). The results showed that the *shi2* mutant had a 4.28-fold increase in eIF4E-bound *LUC* mRNAs compared with the wild type under normal growth conditions, but was 6.7-fold lower in the *shi2* mutant than in the wild type under salt stress (Fig. 9B). Uncapped *LUC* transcripts were also 2.91-fold higher in the *shi2* mutant than in the wild type under normal growth conditions, while they were 3.3-fold lower in the *shi2* mutant than in the wild type under salt stress (Fig. 9B). These results indicate that capped *LUC* mRNAs were more abundant in the *shi2* mutant than in the wild type under normal conditions. However, the capped *LUC* mRNAs were significantly decreased in the *shi2* mutant compared with the wild type under salt stress. Interestingly, the capping pattern of the native *SOT12* revealed no differences between the wild type and *shi2* mutant under both normal and salt stress conditions (Supplementary Fig. S6).

**Fig. 10.**
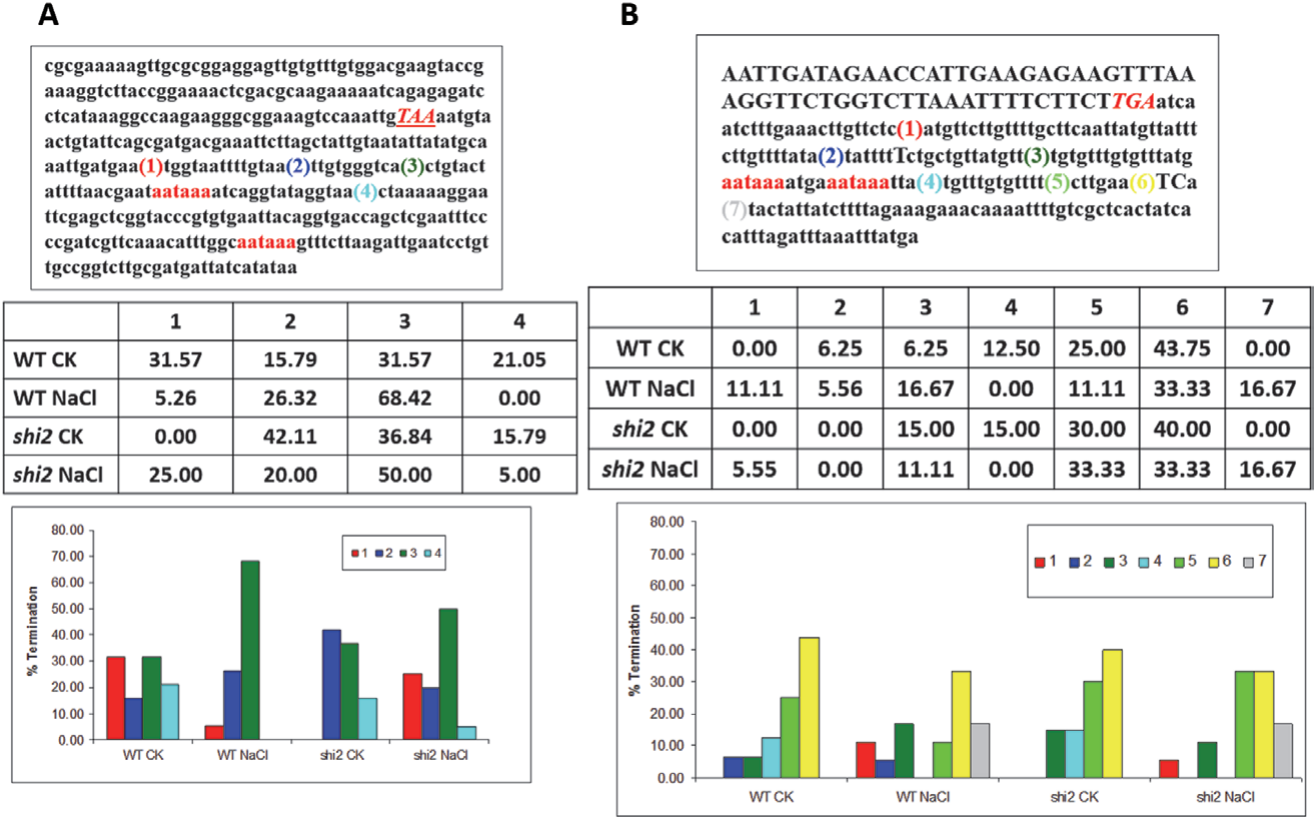
The polyadenylation site selection is altered in the *shi2* mutant. (A) Polyadenylation sites of the *LUC* transgene determined by using 3' RACE. Four detected polyadenylation sites and the ratio of polyadenylation at each site are shown. (B) Polyadenylation sites of the native *SOT12* gene determined by using 3' RACE. Seven detected polyadenylation sites and the ratio of polyadenylation at each site are shown. CK, control without salt treatment; NaCl, 200 mM NaCl treatment for 5 h. (This figure is available in color at *JXB* online.)

The 3' polyadenylation site selection was assessed using 3' RACE. Four termination sites in the *LUC* transgene were found, and the first three sites were located upstream while the fourth site was downstream of the canonical polyadenylation signal sequence AAUAAA (Fig. 10A). Under normal growth conditions, the *shi2* mutant showed preferential selection for sites 2 and 3, while the wild type displayed a relatively even distribution in all four sites, with a slight preference for sites 1 and 3. Under salt stress, both the wild type and *shi2* mutant favored the selection of site 3. However, the *shi2* mutant showed significantly higher selection for site 1 for polyadenylation than the wild type (Fig. 10A). The termination patterns in the native *SOT12* gene were more complex. Seven termination sites were found, with three sites upstream and four sites downstream of the putative polyadenylation signal AAUAAA. Termination patterns were relatively evenly distributed in the *shi2* mutant and wild type under both normal and stress conditions, with slight preference for sites 5 and 6, except that the *shi2* mutant had a notable decrease at site 4 after salt stress treatment (Fig. 10B).

### The *shi2* mutant shows overall reduced molecular response to cold stress

**Fig. 11.**
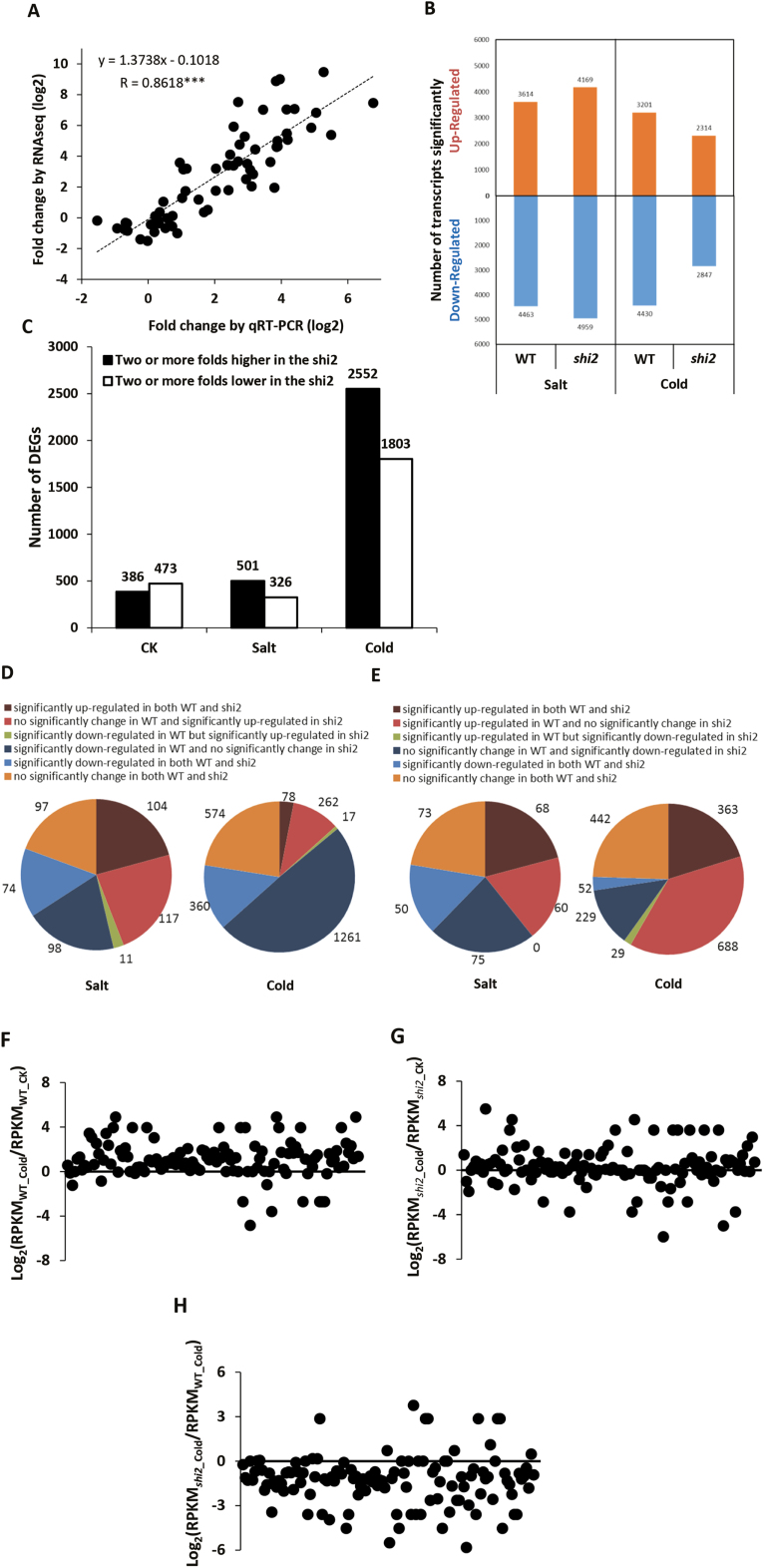
Transcriptome analysis of the wild type and *shi2* mutant. (A) Correlations between RNA-seq data and RT–qPCR data of the16 selected genes (***P<0.001). (B) The numbers of significantly up- and down-regulated genes (≥2-fold change, *P*<0.05, FDR <0.05) in the wild type and *shi2* mutant after salt and cold treatments. (C) The number of ≥2-fold changed (*P*<0.05, FDR <0.05) DEGs between the wild type and *shi2* mutant under normal, salt, and cold stress conditions. (D) and (E) Analysis of DEGs at least 2-fold higher (D) or 2-fold lower (E) in the *shi2* mutant after salt or cold stress. All DEGs were divided into six groups based on their changes in expression levels in the wild type and *shi2* mutant. (F) and (G) Comparison of the expression levels of mitochondria-encoded genes in the wild type (F) or the *shi2* mutant (G) before and after cold stress. (H) Comparison of the expression levels of the mitochondria-encoded genes between the wild type and *shi2* mutant after cold stress. For (F) and (G), each dark dot represents a mitochondria-encoded gene. The horizontal line indicates an equal expression level before and after cold stress. The dot above the line means up-regulation of this gene after cold stress, and the dot below the line means down-regulation of this gene after cold stress. For (H), each dark dot represents a mitochondria-encoded gene. The horizontal line indicates an equal expression level between the wild type and the *shi2* mutant. The dot above the line means the expression level of this gene is higher in the *shi2* mutant than in the wild type, and the dot below the line means the expression level of this gene is lower in the *shi2* mutant than in the wild type. (This figure is available in color at *JXB* online.)

We performed RNA-seq analysis to assess the effects of the *shi2* mutation on genome-wide gene expression. The RNA-seq data were validated by RT–qPCR analysis of 16 genes. Based on the calculation of 64 data sets, the RNA-seq and RT–qPCR are significantly correlated, with *r*=0.8618 and *P*<0.001 (Fig. 11A), indicating that the RNA-seq data are reliable.

Analysis of DEGs between control and treated conditions revealed that there were more DEGs in the *shi2* mutant than in the wild type after salt treatment and fewer DEGs in the *shi2* mutant than in the wild type after cold treatment (Fig. 11B). These findings suggest that the *shi2* mutation resulted in a stronger response to salt stress but a weaker response to cold stress. Analysis of DEGs between the *shi2* mutant and the wild type showed that the *shi2* mutation affected gene expression under normal growth conditions, and cold stress resulted in significantly more DEGs between *shi2* and the wild type than salt stress (Fig. 11C; Supplementary Dataset S1).

Detailed analysis of the RNA-seq data, as shown in Fig. 11D and E, revealed that among the 2552 DEGs with higher expression levels in the *shi2* mutant after cold treatment, 1621 genes (63.5%) were due to a weaker response to cold stress in the *shi2* mutant than in the wild type. Similarly, among the 1803 DEGs which showed lower expression levels in the *shi2* mutant after cold treatment, 1051 genes (58.3%) displayed weaker response to cold stress in the *shi2* mutant than in the wild type. Overall, among the 4355 DEGs between the cold-stressed wild type and the *shi2* mutant, only a small portion (641 genes, 14.7%) of them were caused by their stronger response to cold stress in the *shi2* mutant whereas 61.4% of them (2672 genes) were a result of a weak response to cold stress. In contrast, 41.8% of the DEGs identified between salt-stressed *shi2* and the wild type were genes that responded strongly (346 genes out of 827), and 36.3% of the DEGs (300 genes out of 827) were a result of their weaker response. These results suggest that the *shi2* mutation reduced the magnitude of the response of the cold-responsive genes. For the salt stress response, however, there is not such a clear pattern in the *shi2* mutant.

To further assess the attenuation of the cold response in the *shi2* mutant, 112 mitochondria-encoded genes were analyzed. In the wild type, most of the mitochondria-encoded genes were induced by cold stress (Fig. 11F). However, the number of cold-induced mitochondria-encoded genes was clearly reduced in the *shi2* mutant (Fig. 11G). As a result, the expression levels of most mitochondria-encoded genes in the *shi2* mutant were lower than those in the wild type after cold treatment (Fig. 11H). Together, the RNA-seq data revealed that the *shi2* mutant has an overall attenuated cold stress response with fewer cold-responsive genes and a reduced magnitude of the response of these cold-responsive genes. This attenuated response to cold may account for the hypersensitivity of the *shi2* mutant to low temperature conditions. Although salt stress induced more genes in the wild type and the *shi2* mutant than cold stress (Fig. 11B), the number of DEGs between the wild type and the *shi2* mutant under salt stress was much less than those under cold stress (Fig. 11C), indicating that the impact of the *shi2* mutation is greater on the cold response than on the salt response.

**Fig. 12.**
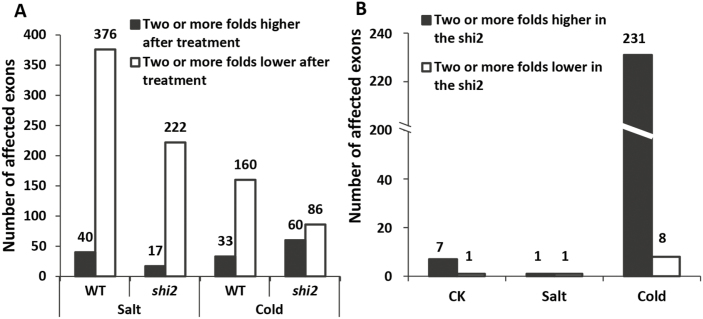
Analysis of differentially expressed exons. (A) The number of significantly up- and down-regulated exons (≥2-fold change, *P*<0.05, *P*adjust<0.1) in the wild type and the *shi2* mutant after salt and cold treatment. (B) The number of ≥2-fold changed (*P*<0.05, *P*adjust<0.1) exons between the wild type and the *shi2* mutant under normal growth conditions, salt stress conditions, and cold stress conditions.

The effect of the *shi2* mutation on mRNA splicing was also examined by using the RNA-seq data based on the expression level of each exon. In the wild type, 376 exons (90.4%) showed a lower expression level after salt stress (fold change >2, *P*<0.05) and 160 exons (82.9%) after cold stress (Fig. 12A). These results indicated that salt and cold stress resulted in the removal of exons from certain mRNAs, which might be a post-transcriptional regulation to generate protein variants with distinct functions in response to these stress conditions. In the *shi2* mutant, the ratio of exons expressed at a lower level (222 exons, 92.9%) and more highly expressed exons (17 exons, 7.1%) after salt stress was very similar to that in the wild type (Fig. 12A). Among them, only two were differentially expressed between *shi2* and the wild type (Fig. 12B). These results indicate that the *shi2* mutation has a minor effect on the mRNA splicing in response to salt stress. On the other hand, cold stress dramatically changed the ratio of exons expressed at a lower level (86 exons, 58.9%) and more highly expressed exons (60 exons, 41.1%) in the *shi2* mutant, compared with 17% and 83% in the wild type, respectively (Fig. 12A). As a result, in the 239 exons that are differentially expressed between the wild type and the *shi2* mutant after cold treatment, 96.7% of them (231 exons) were more highly expressed in the *shi2* mutant, whereas only 3.3% of them (8 exons) were underexpressed in the *shi2* mutant (Fig. 12B). These results suggest that the *shi2* mutation could affect the cold stress response by retaining those exons that should be removed from the mature mRNA in response to cold stress.

## Discussion

In this study, we identified the DEAD-box RNA helicase SHI2 as a repressor for salt-inducible genes. SHI2 also modulates cold stress response by maintaining proper splicing of cold-responsive genes. Our results suggest that *SHI2* is involved in transcription and post-transcriptional processes such as 5' capping and 3' poly(A) site selection.

### SHI2 is involved in maintaining proper splicing of mRNA of cold-responsive genes under cold stress conditions

A *shi2* allele designated as *rcf1* was previously reported to affect the expression of the LUC reporter gene driven by the cold-inducible *CBF2* promoter (Guan *et al.*, 2013). RCF1 functions in maintaining proper pre-mRNA splicing and plays a crucial role in regulating cold-responsive genes (Guan *et al.*, 2013). Consistent with the findings of Guan *et al.* (2013), SHI2 was also found to be involved in pre-mRNA splicing of the cold-responsive genes *COR15A*, *EDR10*, *KIN2*, and *RD22* (Fig. 6). Transcriptomic mRNA splicing analysis further revealed that the *shi2* mutation preferentially affected mRNA splicing under cold stress conditions (Fig. 12), which supports the important role of SHI2 in cold stress response. Meanwhile, the *shi2* mutant displayed a hypersensitive phenotype to LiCl (Fig. 7), which is a well-known inhibitor of RNA processing enzymes (Dichtl *et al.*, 1997), further supporting the role of SHI2 in RNA splicing. Taken together, we propose that the RNA helicase SHI2 is involved in pre-mRNA splicing and affects cold tolerance through maintaining proper mRNA splicing of the genes important for cold response. In addition, an overall attenuated cold stress response in *shi2* (Fig. 11) may contribute to its hypersensitivity to cold.

### SHI2 functions as a repressor for salt-inducible genes

In contrast to attenuated cold response of the *shi2* mutant, the *shi2* mutation displayed a minor effect on mRNA splicing in response to salt stress but increased the number of salt-responsive genes (Figs 4, 11). This suggests that the regulation of salt-responsive genes by SHI2 is unlikely to be due to mRNA splicing, but is more likely to be executed at the transcription level. The *shi2* mutant was isolated based on increased expression of the LUC reporter gene driven by the salt-inducible *AtSOT12* promoter. Both LUC and *AtSOT12* are intronless genes. Therefore, the repression of these genes by SHI2 is clearly independent of its function in mRNA splicing. The C-terminus of SHI2, which does not include the RNA helicase domain, could function as an activator in both yeast and plant cells (Fig. 8), suggesting that SHI2 may interact with and enhance the binding of the general transcription machinery at the promoter region. Both 5' RACE and RT–qPCR experiments revealed that capped *LUC* mRNAs were more abundant in the *shi2* mutant than in the wild type under normal growth conditions (Fig. 9), which resembles our previously reported *shi1* and *shi4* mutants (Jiang *et al.*, 2013). The SHI1–SHI4 complex represses gene transcription by preventing mRNA capping and transition from transcription initiation to elongation (Jiang *et al.*, 2013). Thus, SHI2 may also function as a gene repressor by affecting mRNA capping and transcription initiation.

### SHI2, a splicing factor, may be a component of a ready-for-transcription repressor complex

Inducible gene expression is generally believed to be activated by transcription factors binding to the promoter *cis*-elements, which recruits the general transcription machinery to promote transcription. However, how inducible genes are repressed under non-inducible conditions remains elusive. Our findings suggest that a repressor complex may include the general transcription machinery and other factors such as SHI2 at the promoter to repress the transcription of the inducible genes. Several genome-wide analyses of RNA polymerase occupancy in yeast (Radonjic *et al.*, 2005), human (Guenther *et al.*, 2007), and *Drosophila* (Muse *et al.*, 2007; Zeitlinger *et al.*, 2007; Hendrix *et al.*, 2008) indicate that most protein-coding genes, including those that are inactive but inducible by environmental stimuli or developmental signals, are occupied by a transcription initiation complex. These studies suggest that the inducible genes are repressed but poised for transcription under non-inducible conditions. Our previous studies on SHI1–SHI4 (Jiang *et al.*, 2013) and SHI5/HDA6 (Wang *et al.*, 2017), together with the results of this study, suggest that, under normal growth conditions, the inducible promoters such as the *AtSOT12* promoter may be occupied by a repression complex. This complex may comprise not only the general transcription machinery, but also proteins or enzymes modulating RNA polymerase II (e.g. SHI1 and SHI4) and proteins involved in co-transcriptional processes, such as the splicing factor SHI2. Therefore, we propose that, under normal growth conditions, such a repression complex is berthed at the promoters of stress-inducible genes but poised for transcription. Upon stress, changes to the components within the repressor complex could be modulated by either transcriptional regulation or post-translational modifications of the components, leading to the release of the repressor proteins and the recruitment of the activator proteins into the complex, hence changing the repressor complex into an activation complex for stress-induced gene expression. We postulate that the repressor complex and activation complex share components for general transcription and co-transcriptional processes, thereby ensuring the stress-inducible genes are repressed under normal growth conditions but poised for transcription and can be rapidly induced in response to stresses.

The ability of SHI2 in activating gene expression and the effects of *shi2* mutation on 5' capping, mRNA splicing, and polyadenylation site selection provide compelling evidence that SHI2 is probably associated with transcription machinery that regulates transcription of stress-inducible genes under normal and stressed conditions. In our future work, identification of the SHI2-associated proteins will help our understanding of the SHI2-associated repressor complex for stress-inducible gene regulation.

## Supplementary data

Supplementary data are available at *JXB* online.

**Fig. S1.** The wild type and the *shi2* mutant respond similarly to NaCl and mannitol.

**Fig. S2.** Genetic complementation of the *shi2* mutant with the T-DNA mutant (SALK_146567).

**Fig. S3.** Molecular complementation of the *shi2* LiCl-sensitive phenotype.

**Fig. S4.** Protein sequence analysis of SHI2.

**Fig. S5.** Detection of intron retention of *COR15A* and *KIN2/COR6.6* transcripts.

**Fig. S6.** Detection of 5' capping of the native *AtSOT12* mRNA using 5' RACE.

**Table S1.** Primers for constructs.

**Table S2.** RT–qPCR primers used in Figs 4 and 6.

**Table S3.** Primer information for 5' and 3' RACE PCR and RT–qPCR.

**Table S4.** Primers used in RT–qPCR for RNA-seq validation.

**Dataset S1.** List of genes expressed in the *shi2* mutant.

erz523_suppl_Supplementary_file001

erz523_suppl_Supplementary_file002
