# Supplementary material for: The DEAD-box RNA helicase SHI2 functions in repression of salt-inducible genes and regulation of cold-inducible gene splicing
Source: J Exp Bot. 2019 Nov 20;71(4):1598–613. doi: 10.1093/jxb/erz523 (PMC7242002; doi:10.1093/jxb/erz523)
Supplement: erz523_suppl_Supplementary_file001 [file erz523_suppl_supplementary_file001.pdf]

Supplemental figures

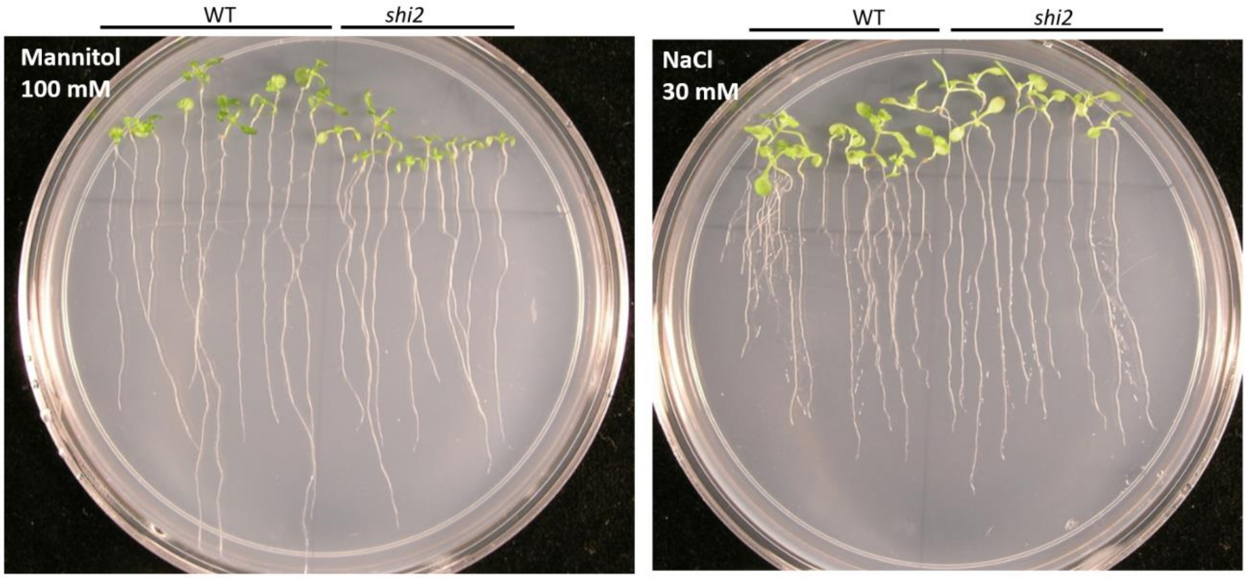

Figure S1. The wild type and the *shi2* mutant respond similarly to NaCl and mannitol
Six-day-old seedlings were transferred from ½ MS medium to the ½ MS medium supplemented with 100 mM mannitol/ 30 mM NaCl and allowed to grow for another 6 days.

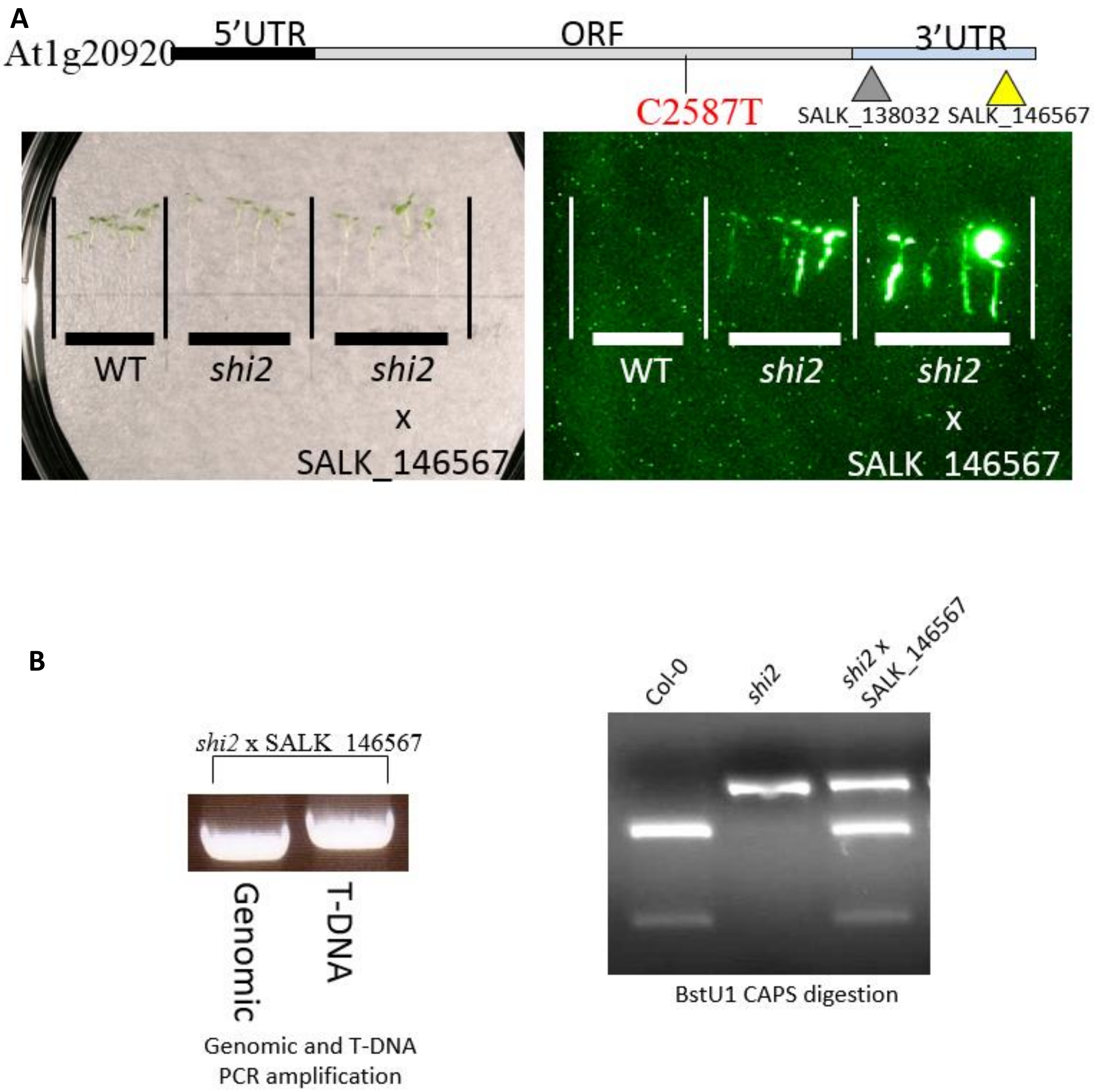

Figure S2. Genetic complementation of the *shi2* mutant with the T-DNA mutant (SALK_146567). (A) Schematic representation of *SHI2* gene with the *shi2* mutation and the T-DNA insertions. LUC imaging of WT, *shi2* and *shi2* x *SALK_146567* (F1) seedlings is shown in the right panel. (B) PCR-based genotyping of the *shi2* x *SALK_146567* F1 seedlings to confirm the presence of both *shi2* mutation and the T-DNA insertion.

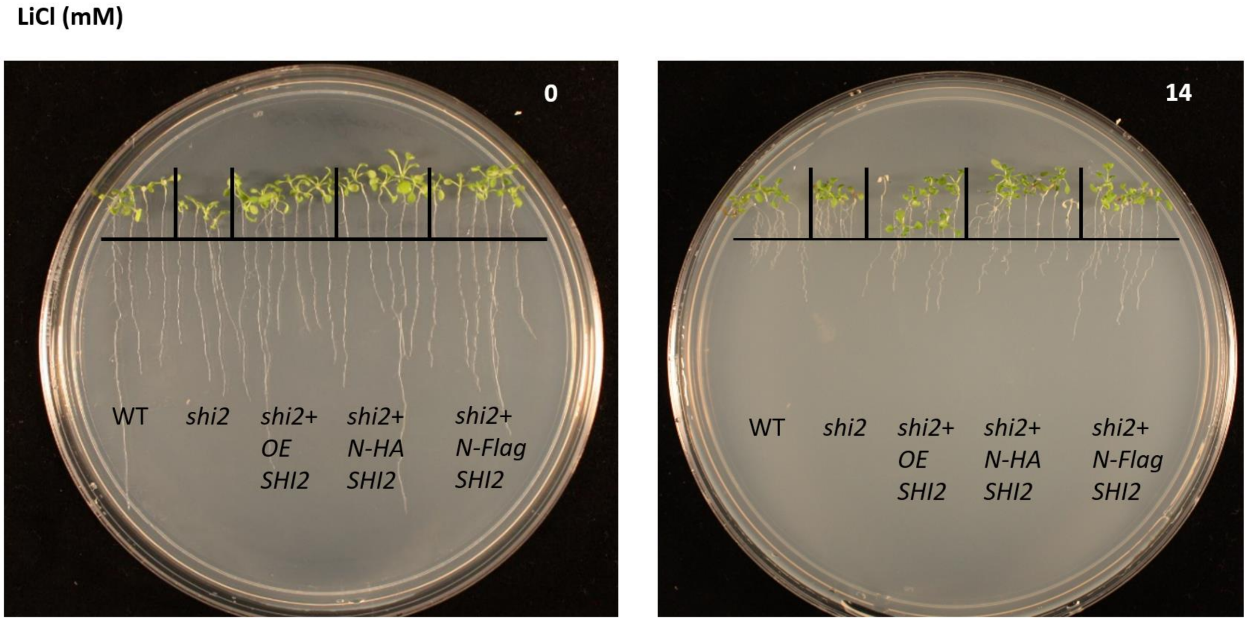

Figure S3. Molecular complementation of *shi2*. 7-day-old seedlings grown vertically on normal ½ MS agar (1.2%) medium were transferred to ½ MS agar (1.2%) medium without or with 14 mM LiCl, and pictures were taken 12 days after the transfer.

A

```
                                                              Section 1
            (1)  1        10        20        30        40            54
PRP5        (1)  ------------------------------------------------------
SHI2        (1)  MEVEKSKYRSEDLDVVEEEADLKKSRRDRDRSNERKKDKGSEKRREKDRRKKRV
Consensus   (1)
                                                              Section 2
           (55)  55   60        70        80        90               108
PRP5        (1)  ------------------------------------------------------
SHI2       (55)  KSSDSEDDYDRDDDEEREKRKEKERERRRDKDRVKRRSERRKSSDSEDDVEEE
Consensus  (55)
                                                              Section 3
          (109)  109       120       130       140       150         162
PRP5        (1)  -------------------------------------METIDSKQNINRESLLE
SHI2      (109)  DERDKRRVNEKERGHREHERDRGKDRKRDREREERKDKEREREKDRERRERERE
Consensus (109)                                        E    K    RE   E
                                                              Section 4
          (163)  163    170       180       190       200            216
PRP5       (18)  ERRKKLAKWKQK-------KAQFDAQKEHQTSRN--------------------
SHI2      (163)  EREKERVKERERREREDGERDRREREKERGSRRNRERERSREVGNEESDDDVKR
Consensus (163)  ER K   K K K       K   D  KE  S RN
                                                              Section 5
          (217)  217         230       240       250       260       270
PRP5       (45)  DIVTNSLEGKQTTEKFTER-----------------------------------
SHI2      (217)  DLKRRRKEGGERKEKEREKSVGRSSRHEDSPKRKSVEDNGEKKEKKTREEELED
Consensus (217)  DI     EG    EK  EK
                                                              Section 6
          (271)  271      280       290       300       310          324
PRP5       (64)  -QERVKEELRKRKN-------EFRKSDEPVSVKPSKKKSKRSKVKKKISFDFSD
SHI2      (271)  EQKKLDEEVEKRRRRVQEWQELKRKKEEAESESKGDADGNEPKAGKAWTLEGES
Consensus (271)   Q KL EEL KRK          RK DE  S           K  K  S D
                                                              Section 7
          (325)  325  330       340       350       360              378
PRP5      (110)  DDDS---------EIGVSFRSK-------------------E--HIQKAPEHDN
SHI2      (325)  DDEEGHPEEKSETEMDVDEETKPENDGDAKMVDLENETAATVSESGGDGAVDEE
Consensus (325)  DDD          EI V   SK                          A   D
                                                              Section 8
          (379)  379        390       400       410       420          432
PRP5      (134)  EKDPLDEFMTSLKE--------------------EKMSNSKGMYDR--------
SHI2      (379)  EIDPLDAFMNTMVLPEVEKFCNGAPPPAVNDGTLDSKMNGKESGDRPKKGFNKA
Consensus (379)  E DPLD FM SL                      D   N K   DR
                                                              Section 9
          (433)  433    440       450       460       470            486
PRP5      (160)  -GDILDVEDQLFELGG-TDDEDVEDNTDNSNIAKIAKLKAKKRVKQIYYSPEEL
SHI2      (433)  LGRIIQGEDSDSDYSEPKNDDDPSLDEDDEEFMKRVKKTKAEKLSLVDHSKIEY
Consensus (433)   G II   ED   D     DDD    D      K  K     KL  I HS  E
```

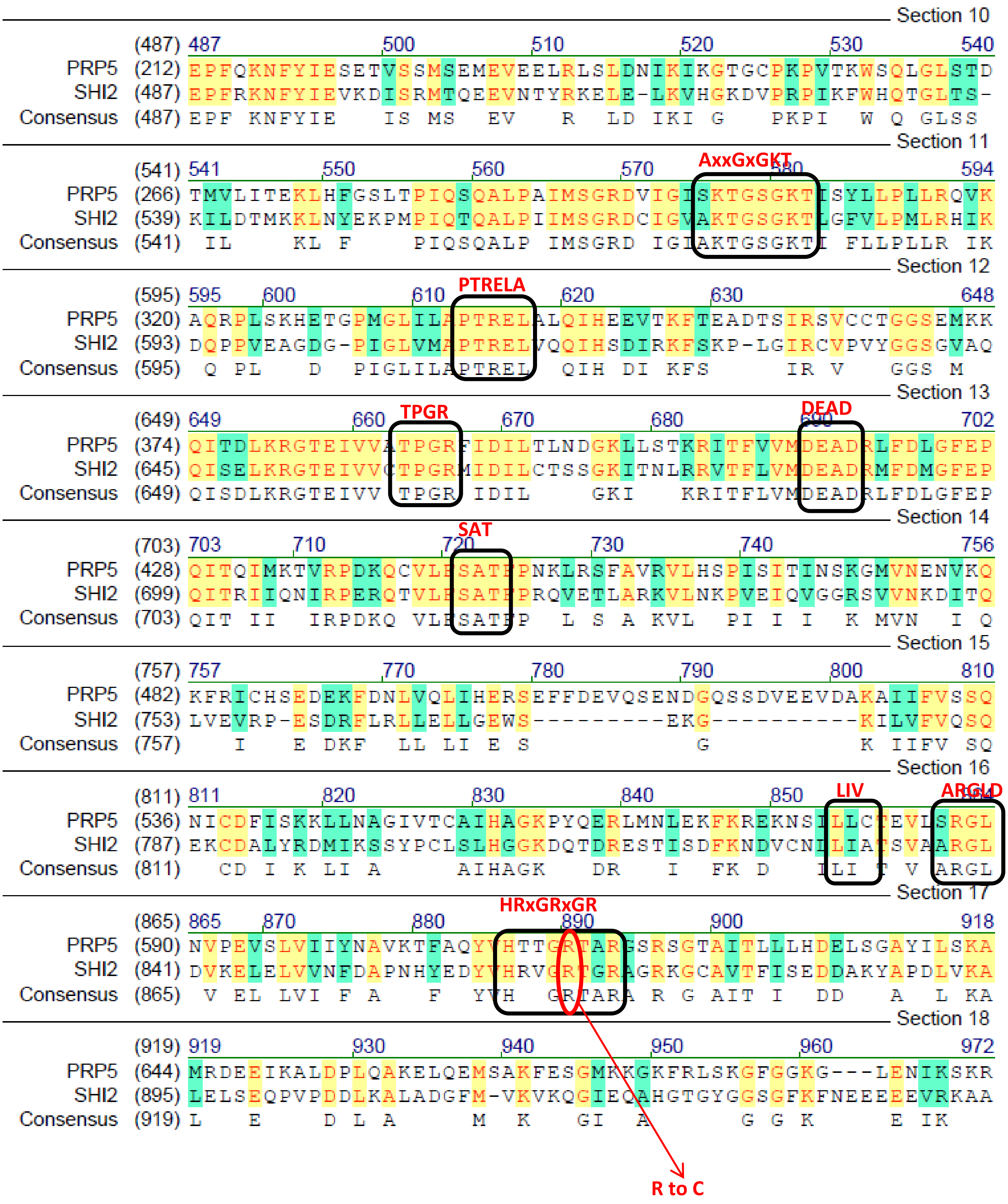
Section 10
Section 11
Section 12
Section 13
Section 14
Section 15
Section 16
Section 17
Section 18
PRP5
SHI2
Consensus
AxxGxGKT
PTRELA
TPGR
DEAD
SAT
LIV
ARGLD
HRxGRxGR
R to C

```
                                                                    Section 19
                (973) 973          980                990                1000               1010                        1026
         PRP5   (695) EEAQNKDLELKKNDKRSDDLEKKISNPREGHDSVS-------------------
         SHI2   (948) KKAQAKEYGFEEDKSDSEDENDVVRKAGGGEISQQQATFAQIAAIAAAAKAAAA
    Consensus   (973)   AQ KD          SDD     I     G  S
                                                                    Section 20
               (1027) 1027           1040       1050       1060       1070       1080
         PRP5   (730) --------------------ESSALIPR---------------------LNYEL
         SHI2  (1002) APVSAPVTANQLLANGGGLAAMPGVLPVTVPTLPSEGAGRAAAMVAAMNLQHNL
    Consensus  (1027)                        ALIP                     LNH L
                                                                    Section 21
               (1081) 1081     1090       1100       1110       1120         1134
         PRP5   (743) FKESTDGS-IIFYAKVYINDLPQIVRWEATKNTTLLFIKHETGCSITNKGKFYP
         SHI2  (1056) AKIQADAMPEHYEAELEINDFPQNARWKVTHKETLGPISEWTGAAITTRGQFYP
    Consensus  (1081)  K   DA     F A L IND PQ  RW  T    TL   I    TG AIT KG FYP
                                                                    Section 22
               (1135) 1135  1140       1150       1160       1170         1188
         PRP5   (796) EGKEPKNENDEPKLYLLIEGQDEKDIQLSIELLEQKVKEGVVKAASLSLKSTKY
         SHI2  (1110) TGRIPGP--GERKLYLFIEGPSEKSVKHAKAELKRVLEDITNQAMSSLPGGASG
    Consensus  (1135)  GK P      E KLYL IEG  EK I  A   L   L D     A S
                                                                    Section 23
               (1189) 1189193
         PRP5   (850) -----
         SHI2  (1162) RYSVL
    Consensus  (1189)
```

B

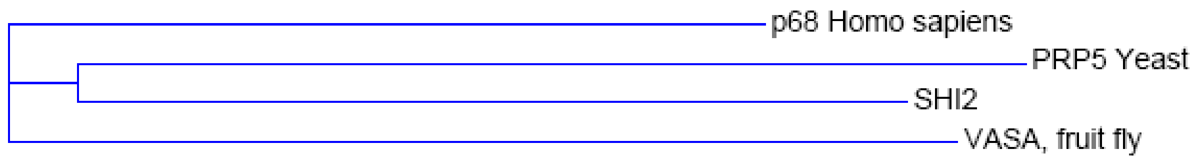

Figure S4. Protein sequence analysis of SHI2

(A) Sequence analysis of the Arabidopsis DEAD box RNA helicase SHI2 showing high similarity with the yeast DEAD box RNA helicase PRP5. The conserved motifs in RNA helicases are shown, and the amino acid substitution in the *shi2* mutant is shown. (B) Phylogenetic tree of DEAD box RNA helicase from different species (Homo-Sapiens, *Saccharomayces*, *Arabidopsis*, and Fruit Fly).

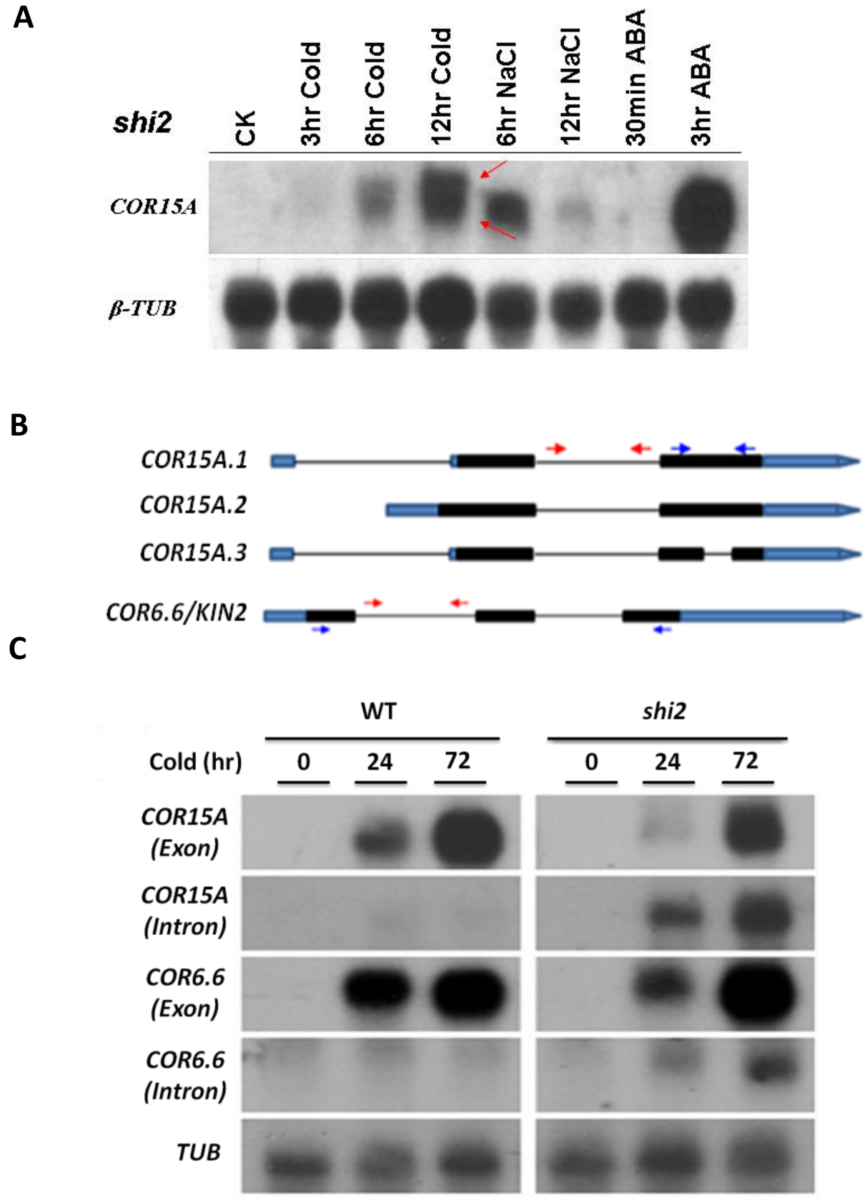

Figure S5. Detection of intron retention of *COR15A* and *COR6.6* transcripts. (A) Northern blot detecting mal-spliced *COR15A* transcripts under cold stress condition. (B) Gene structure of the Arabidopsis *COR15A* and *COR6.6* genes. Primers were designed from exon and intron regions of the genes to determine intron-containing mRNA. (C) Detection of intron-containing *COR15A* and *COR6.6* transcripts using Northern blot. Total RNA (20 µg) from 7-day-old seedlings were subjected to RNA hybridization with probes from coding and non-coding regions of *COR15A* and *COR6.6*. Images were obtained from the same membrane with same hybridization conditions and X-ray film exposure time.

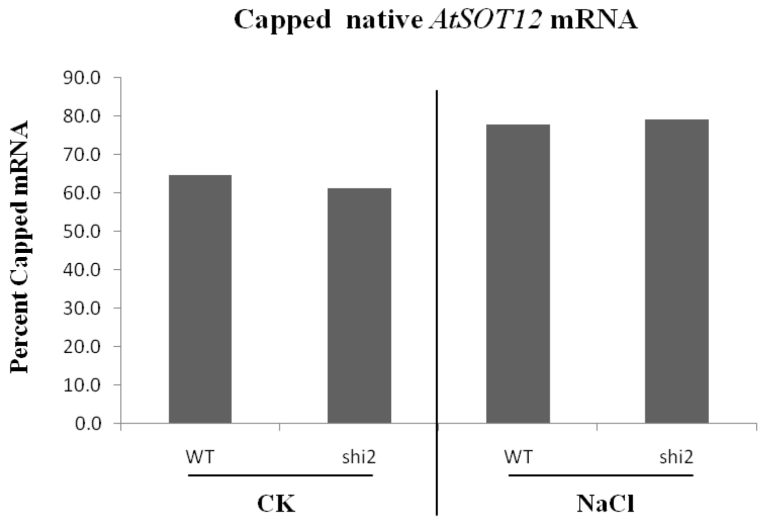

Figure S6. Detection of 5'capping of the native *AtSOT12* mRNA using 5' RACE. The capping pattern of the native *AtSOT12* revealed no difference between wild type and the *shi2* mutant.

Supplemental tables

Table S1. Primers for constructs

Primer info	Primer Sequence	Digestion
SOT12-LUC construct Forward	CCCCCCGGGGAAGGTTTCCACCTTCACACTC	SmaI
SOT12-LUC construct Reverse	AAAACTGCAGTGTTGAGACTTGAGAGATCGATCA	PstI
pENTR1A-SHI2 Forward	ACGCGTCGAC ATGGAGGTAGAGAAATCCAAGTATAGGA	SalI
pENTR1A-SHI2 Reverse 1 (No Stop Codon)	ATAAGAATGCGGCCGCAGGACTGAGTATCTCCCGGATG	NotI
pENTR1A-SHI2 Reverse 2 (Stop Codon)	ATAAGAATGCGGCCGCTTAAAGGACTGAGTATCTCCCGGAT	NotI
SHI2-GUS Forward	CGGAATTCCGGCTTAACTCTGTTCAGATCCTG	EcoRI
SHI2-GUS Reverse	CGGGATCCCCTAAATTCAGCTCCGATAACACA	BamHI
pENTR1A-SHI2 C-Terminal Delete 1 Forward	ACGCGTCGACATGGAGGTAGAGAAATCCAAGTATAGGA	SalI
pENTR1A-SHI2 C-Terminal Delete 1 Reverse	ATAAGAATGCGGCCGCACAGCCCTTACGCCCTGC	NotI
pENTR1A-SHI2 N-Terminal Delete 2 Forward	ACGCGTCGACCTCCCTATCATCATGAGCGGTC	SalI
pENTR1A-SHI2 N-Terminal Delete 2 Reverse	ATAAGAATGCGGCCGCAGGACTGAGTATCTCCCGGATG	NotI
SHI2c Forward	ACGCGTCGACGCTGTGACATTTATCTCCGAGGAT	SalI
SHI2c Reverse	ATAAGAATGCGGCCGCAGGACTGAGTATCTCCCGGATG	NotI
TransActivation SHI2C Forward	CCCCCGGGGGCTGTGACATTTATCTCCGAGGAT	XmaI
TransActivation SHI2C Reverse	ACGCGTCGACTTAAAGGACTGAGTATCTCCCGGAT	SalI
TransActivation ARF5M Forward	CCCCCCGGGTGGAATCAGCGATTTGGATCC	XmaI
TransActivation ARF5M Reverse	ACGCGTCGACGCTTGAAGATGTACCAGTGCCT	SalI
Yeast SHI2 Comp F	CGGGATCCATGGAGGTAGAGAAATCCAAGTATAGG	BamHI
Yeast SHI2 Comp R	CGGAATTCTTAAAGGACTGAGTATCTCCCGG	EcoRI

Table S2. RT-qPCR primers used in Fig. 4 and Fig. 6

Gene ID	Primer Name	Primer Sequence
AT2G42540 COR15A	qCOR15AF	ACCTCAACGAGGCCACAAAGAAAG
	qCOR15AR	CGCTTTCTCACCATCTGCTAATGC
	COR15A inF	CTACTTTTGGATATGTTTTGAAACCTT
	COR15A inR	CCTCTTTTGTTTTATCCGTCACG
AT2G20440 COR47	qCOR47F	AGTTAGCTGCGGAGCACGAG
	qCOR47R	CATCGCTCGAAGAGGAAGAAG
	COR47 inF	AGCCTAGTGTCATCGAAAAGC
	COR47 inR	CATGATCTCTTATCAACCATTTCG
AT5G15960 KIN1	qKIN1F	GCAAAGCTGAGGAGAAGAGC
	qKIN1R	CCGCATCCGATACACTCTTTCCC
	KIN1 inF	ACCGCTGGCAAAGCTGAG
	KIN1 inR	CAACTGTTTTCAAAGAGTTATAGCTTC
AT5G15970 KIN2	qKIN2F	GCCGCTGGCAAAGCTGAG
	qKIN2R	TTTCCCGCCTGTTGCGCGGA
	KIN2 inF	GTCAGAGACCAACAAGAATGCC
	KIN2 inR	CTATCAGTACTCTGTTCTAAGAGAG
AT1G20450 ERD10	qERD10F	AGCACAAGACTCAGATCTCTGAACC
	qERD10R	TTCTTCATCACTCGAAGAGGAAGAAG
	ERD10 inF	CAAGTCTCCTCGACAAACTCCAC
	ERD10 inR	GAGTTTGTTTTCAAGATTTAACCAAG
AT5G25610 RD22	qRD22F	GGTTCATTCATGTAGTGGCGATTGC
	qRD22R	TTCTCGTCGGTAAAATCGAAAGTCAA
	RD22 inF	CTCAAAACGAACAAAACATGATTAC
	RD22 inR	AGTGTTTGGTAAAGCAGTGCTCC
AT5G05410 DREB2A	qDREB2aF	TCCCCTATAGATGGCAGTTTATGATC
	qDREB2aR	CGGTCTCGTTATACTCTTTCCATCTC
	DREB2a inF	CTCGTCAATTGGTGATTCTAGGG
	DREB2a inR	AGAACACAAGCTGAAACATAAGGC
AT5G52310 RD29A	qRD29AF	CTTGATGGTCAACGGAAGGT
	qRD29AR	CAATCTCCGGTACTCCTCCA
	RD29A inF	CCACCACTCAACACACACC
	RD29A inR	GTTTAAGTAAATTCAGAGGATTGTC
AT2G03760 SOT12	qSOT12F	AAGGACTTTGGCACACACAAGC
	qSOT12R	AAGAAACTGGAAACTTGTGTCGG
AT5G09810 ACT7	qACT7F	GTTGCCATTCAGGCCGTTCTTTC
	qACT7R	CAGAATCGAGCACAATACCGGTTG

Table S3. Primer information for 5’ and 3’ RACE PCR and RT-qPCR.

**Primer name**	**Sequence**
LUC 3’ RACE	GTTTTGGAGCACGGAAAGACG
LUC 3’ RACE nested 1	CGTGGATTACGTCGCCAGTC
LUC 3’ RACE nested 2	GCCAAGAAGGGCGGAAAGTC
SOT12 3’ RACE	TTGCCAAATGGAATAGAGACTAAAAC
SOT12 3’ RACE nested 1	GGAGAGATACTTTGAGTGAGTCATTGG
SOT12 3’ RACE nested 2	AGTGAGTCATTGGCAGAGGAAATTG
LUC 5’ RACE	GGTTGGCAGAAGCTATGAAAC
LUC 5’ RACE 2	GTTCACCTCGATATGTGCATCTGT
LUC 5’ RACE nested 1	GAAGAGATACGCCCTGGTTCCTGG
LUC 5’ RACE nested 2	GCAGTTGCTCTCCAGCGGTT
SOT12 5’ RACE	TCAGAGCTCTTGTTTCTTGTGTCAGA
SOT12 5’ RACE nested 1	TTCATCTCCCAAGTAAGCAGGAAC
SOT12 5’ RACE nested 2	AGCAGGAACTGATGATGATGATGA
RACE adaptor dT	CTGATCTAGAGGTACCGGATCCTTTTTTTTTTTTTTTTT
RACE adaptor	CTGATCTAGAGGTACCGGATCC
Real-Time LUC F	TGGAGAGCAACTGCATAAGG
Real-Time LUC R	GTTCACCTCGATATGTGCATCTGT
Real-Time SOT12 F	GGAGAGATACTTTGAGTGAGTCATTGG
Real-Time SOT12 R	GCAAAACAAGAACATGAGAACAAG
Actin2 F	ACACTGTGCCAATCTACGAGGGTT
Actin2 R	ACAATTTCCCGCTCTGCTGTTGTG

Table S4. Primers used in RT-qPCR for RNA-seq validation.

Gene	Forward and Reverse primer sequence
CBF3	CGACGACGGATCATGGCTTC<br>CTCCATAACGATACGTCGTCATC
COR15A	AGATGGTGAGAAAGCGAAAGACTAC<br>GAACTCTGCCGCCTTGTTTG
COR47	TTCACCAGCTGTCACGTCCA<br>CTTCTCCTCCGGATGTTCCA
RAS1	TCGTGGCCCAGTTTCTATCTCA<br>CGAGATCCGGTCTTTCTGGTG
ERF12	CCGCAAATAACCAACCCCAC<br>CTGTGTTAGGCGAGGAGGTGAAG
ERD7	TCGTCCTACCAAAGAGATCTCACA<br>TTCCTTCGCTTCCTCTGACAC
COR27	GGATTAACGGAAGGCACGGT<br>GCTGCTTGAGACTAGAGGCTGAG
COR78	GGTGGGCTTTGGTGACGAGT<br>AGCCCATCGGAGAATTCTTGTC
LTI30	TCACATCAAACTGGGACTAACACG<br>GGTGAACAACGCCAGTATTACCA
SOT12	ATCATCATCAGTTCCTGCTTACTTG<br>AGCTTTTAACCAAGTGGTACCTGA
PSBA	ATCGCATTCATTGCTGCTCC<br>TCACGACCCATATAACAAGCTACAC
CA1	TTCAGCCTTTGAAGATCAATGTG<br>CCAAAGCTCAAAAGCACCCT
BIN2	TTGGAGACTGGAGAAACCGTG<br>CGATACAAGCTCTCAGGGACATACT
LHCA3	TCCAAACCGGTGTGATTCCA<br>CCGGCCAAACCCTTCTCTAAC
CRB	CGTCCTGTCTACATCTACGGTCC<br>CCAAGCACGTTGAGAAAGGC
ROC4	CATTCAATGGCTTCTTCGTCTTC<br>GAAAATTGCTTAATGGGTGATGC
ACTIN2	CCGAGTATGATGAGGCAGGTC<br>CCCATTCATAAAACCCCAGC
